# Curvy Surface Reconstruction

**DOI:** 10.1002/advs.202516891

**Published:** 2026-01-04

**Authors:** Chen Shang, Haoyu Qi, Zhigang Wang, Keyu Meng, Zeye Liu, Zeng Meng, Yu Yang, Jianjun Wang, Shan Jiang

**Affiliations:** ^1^ College of Mechanical and Electrical Engineering Shaanxi University of Science and Technology Xi'an China; ^2^ Hangzhou Institute of Technology Xidian University Hangzhou China; ^3^ National Key Laboratory of Strength and Structural Integrity Aircraft Strength Research Institute of China Xi'an China; ^4^ School of Electronic and Information Engineering Changchun University Changchun China; ^5^ Department of Cardiac Surgery Peking University People's Hospital Peking University Beijing China; ^6^ School of Civil Engineering Hefei University of Technology Hefei China; ^7^ State Key Laboratory of Electromechanical Integrated Manufacturing of High‐Performance Electronic Equipments Xidian University Xi'an China

**Keywords:** curvy surface reconstruction, advanced measurement methods, AI reconstruction algorithms, conformal design and fabrication

## Abstract

The physical world around us is inherently curvy, dynamic, and variable, yet modern industrial civilization is grounded in the planar, rigid paradigms of science and technology. This fundamental disconnect between two‐dimensional (2D) techniques and three‐dimensional (3D) realities significantly restricts our ability to fully perceive and to understand the complexity of real‐world objects. Over the past several decades, driven by application demands across various industries, advancements in high‐speed, high‐accuracy, and high‐resolution sensors, as well as ever‐increasing AI algorithms and computational power, curvy surface reconstruction that can reconstruct continuous, smooth geometrical and physical fields from discrete data by algorithms and mathematics have experienced tremendous developments. However, previous reviews in this field have primarily focused on geometric shapes, optical measurement techniques, or reconstruction algorithms, leaving a comprehensive overview that integrates both geometric and physical dimensions still lacking. Here, for the first time, we bridge this gap by expanding the scope from special curvy imaging to general curvy reconstruction incorporating physical fields, with a particular emphasis on measurement techniques, especially the emerging opportunities from advanced techniques. Initially, a brief overview starts with introducing the theoretical underpinnings and primary issues of curvy surface reconstruction. Next, an in‐depth discussion of the main non‐contact and contact measurement methods is presented, detailing their operational principles, progress, merits and demerits, and future efforts. Following that, several reconstruction algorithms and their applications are discussed. Finally, our insights on the ongoing challenges and opportunities in this field are summarized.

## Graphical abstract

This review introduces the concept of “general curvy surface reconstruction,” which incorporates both geometric and physical dimensions, representing an upgrade over conventional geometric reconstruction that focuses solely on spatial relationships. Particular emphasis is placed on the emerging opportunities enabled by advanced measurement techniques, rapidly evolving AI algorithms, and increasing computational power.

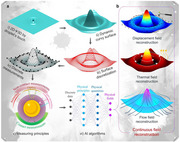

## 1 Introduction

The world is composed of curvy surfaces. Research on curvy surfaces is crucial because it helps us understand and design systems that interact with the natural world, which is inherently irregular and dynamic. By studying these surfaces, we can advance applications in diverse fields like soft robotics [1, 2, 3, 4, 5], electromagnetic cloaks [6, 7, 8, 9, 10], and architecture [11, 12, 13, 14, 15]. Essentially, this research enhances how we build and interact with the world around us by adapting to and leveraging its inherent curvature and variability. With the continuous exploration of the world's true nature and a growing emphasis on human‐centered comfort and customization, 3D curvy applications have emerged as an inevitable trend. However, current industrial civilization is based on metal‐based and silicon‐based planar, rigid science and technology. The conflict between traditional 2D techniques and emerging 3D curvy applications, such as wearable healthcare [16, 17, 18, 19, 20], autonomous driving [21, 22, 23, 24, 25], and conformal printing [26, 27, 28, 29, 30, 31], is becoming increasingly prominent. Transitioning from 2D to 3D in technical and design contexts presents significant challenges, including geometric complexity, material behavior, alignment and calibration, and the exponentially increasing computational load. This shift is not merely about adding an extra dimension but involves navigating a whole new level of geometric complexity and physical principles. Curvy surface reconstruction plays a vital role in addressing these challenges. It involves creating or approximating a smooth, continuous surface that embodies the geometrical and physical characteristics of an object, derived from a set of discrete data points or samples using various reconstruction algorithms [32]. The term “curvy” emphasizes that the surface is no longer flat or planar, but instead captures the intricate, smooth curves and undulations inherent in the object's geometry.

Curvy surface reconstruction primarily involves two steps: (i) the precise measurement of the (*x*, *y*, *z*) coordinates of discrete points on an object's surface and (ii) the rational connection of these points through algorithms to reconstruct a smooth, continuous surface. The measurement results may be viewed as a depth (or range) map, where *z* is a function of the position (*x*, *y*) in a Cartesian coordinate system, and can be expressed in a digital matrix form {*z_ij_* = (*x_i_*, *y_i_*), *i* = 1, 2, …, *L*; *j* = 1, 2, …, *M*}. A more general curvy surface reconstruction system can also capture scalar values, such as surface reflectance, for each point on the nonplanar surface. The result is a point cloud {*P_i_* = (*x_i_*, *y_i_*, *z_i_*, *f_i_*), *i* = 1, 2, …, *N*}, where *f_i_* represents the value at the *i*‐th surface point in the data set. Likewise, a color surface can be represented as {*P_i_* = *x_i_*, *y_i_*, *z_i_*, *r_i_*, *g_i_*, *b_i_*}, *i* = 1, 2, …, *N*}, where the vector (*r_i_*, *g_i_*, *b_i_*) represents the red, green, and blue color components associated with the *i‐*th point. Spectral surface properties may also be described by higher‐dimensional vectors. Notably, curvy surface reconstruction typically refers to the geometric reconstruction of surface shape, which is the special definition of curvy surface reconstruction, primarily applied in 3D modeling [33, 34, 35], computer graphics [36, 37, 38], and virtual reality [39, 40, 41]. This is often termed curvy surface imaging. In contrast, when surface physical fields are also reconstructed, the process follows a more general definition, commonly used in applications such as optical cloaking [42, 43, 44], wearable health monitoring [45, 46, 47], and wind tunnel testing [48, 49, 50]. This discussion focuses on general curvy surface reconstruction, where, unlike special geometric reconstruction that emphasizes spatial relationships, reconstruction algorithms must also incorporate physical principles. These principles account for material properties, environmental influences, and application‐specific constraints, enabling more accurate and functional reconstructions. By integrating geometric and physical data, general curvy surface reconstruction enhances the realism and applicability of 3D representations across multiple disciplines.

**FIGURE 1:**
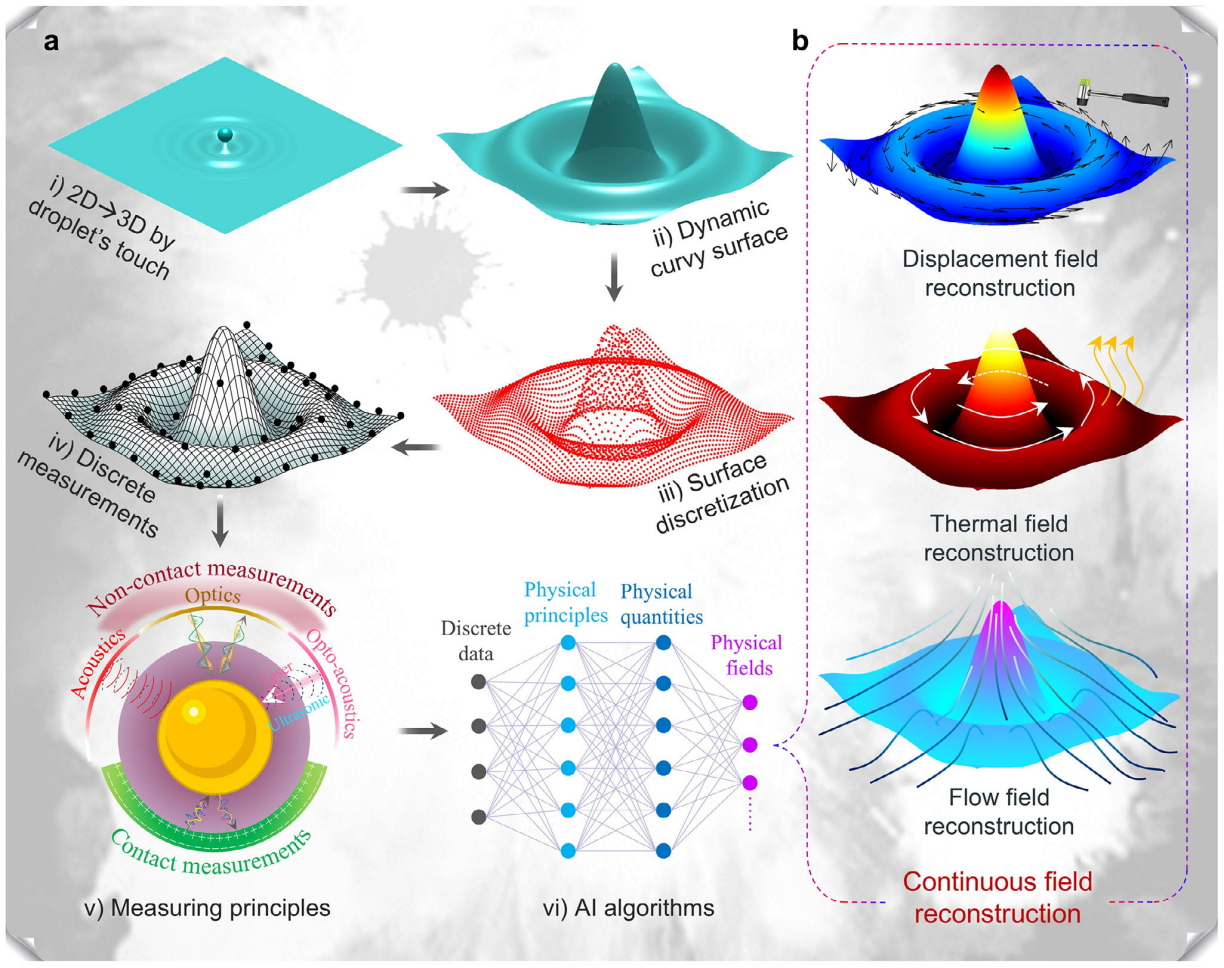
Principle and application of general curvy surface reconstruction. Unlike the traditional “special curvy surface reconstruction” that emphasizes spatial relationships, the general curvy surface reconstruction should simultaneously capture geometric and physical information. (a) A panorama of curvy surface reconstruction, exemplified by the sombrero surface. It starts with the key issue of transitioning from 2D to 3D by droplet's touch, followed by surface discretization and discrete data collection through precise measurement methods, and culminates in the rational connection of points based on reconstruction algorithms and physical principles. (b) Several examples of various applications, including continuous reconstruction of displacement, thermal, and flow fields.

For special curvy surface imaging, the principal methods for obtaining precise measurements of discrete points are mostly light‐based techniques, such as 3D scanning [51, 52, 53] and photogrammetry [54, 55, 56]. However, for general curvy surface reconstruction, measurement methods can be categorized based on operational modes into non‐contact and contact measurements. Moreover, based on their underlying physical principles, non‐contact measurements can be further divided into optical [57, 58, 59, 60, 61], acoustic [62, 63, 64, 65, 66, 67, 68, 69], and photoacoustic measurements [70, 71, 72, 73, 74, 75, 76, 77]. On the other hand, contact measurements can be further categorized into sensitive coatings [78, 79, 80, 81, 82], flexible electronics [83, 84, 85, 86, 87], and flexible fibers [88, 89, 90, 91, 92]. Early reconstruction algorithms relied on mathematical models, later evolving into numerical methods such as finite element analysis [93, 94, 95, 96, 97, 98] and inverse finite element methods (iFEM) [99, 100, 101, 102, 103, 104, 105, 106, 107, 108]. Currently, state‐of‐the‐art approaches leverage artificial intelligence models for more efficient and accurate reconstructions [109, 110, 111, 112, 113, 114]. Figure 1 gives a panorama of curvy surface reconstruction, exemplified by the sombrero surface. In Figure 1a, it starts with the key issue of transitioning from 2D to 3D by droplet's touch, followed by surface discretization and discrete data collection through precise measurement methods, and culminates in the rational connection of points based on physical principles and reconstruction algorithms. Finally, Figure 1b showcases several examples of various applications, including continuous reconstruction of displacement, thermal, and flow fields.

Curvy surface reconstruction enables accurate digital representation and manipulation of complex, curvy objects, bridging the gap between physical, irregular shapes and their computational representations. This capability enhances the feasibility and efficiency of advanced applications across diverse fields. By reconstructing curvy surfaces from real‐world data, precise models can be created for medical imaging [64, 65, 66], manufacturing [115, 116, 117, 118, 119, 120], and simulation [121, 122, 123, 124, 125, 126]. In industries like aerospace or automotive engineering, it facilitates the design of aerodynamic surfaces [127, 128, 129]. For bioengineering, it aids in modeling intricate, adaptable structures [130, 131, 132, 133, 134]. In recent years, driven by application demands across various industries, advancements in high‐speed, high‐accuracy, and high‐resolution sensors, as well as ever‐increasing AI algorithms and computational power, research on curvy surface reconstruction has experienced tremendous developments. While numerous reviews have been published, most focus on geometric shape reconstruction and optical measurement techniques [33, 34, 57, 58, 59]. However, a comprehensive review from the perspective of physical field reconstruction, along with in‐depth insights into diverse measurement techniques, is still lacking. This gap has become especially significant with the emergence of advanced measurement technologies, such as flexible electronics and flexible fibers, which introduce new opportunities for curvy surface reconstruction. This review aims to address this gap and inspire researchers across disciplines. Toward this end, we summarize state‐of‐the‐art advances in various measurement techniques for curvy surface reconstruction, highlighting their operational principles, applications, advantages, limitations, and future directions. We then present several representative reconstruction methods, with special attention given to their existing applications rather than the details of the algorithms. Finally, we close with our perspectives on the remaining challenges and emerging opportunities. Table 1 provides an overview of the critical factors in curvy surface reconstruction, including their classification, advantages, limitations, related figures in this work, and challenges, and also serves as a concise framework and logical summary of the entire review. It should be noted that curvy surface reconstruction is envisioned not as a summary of past research, but rather as a forward‐looking direction for future exploration, aiming to extend current measurement‐based approaches toward integrated geometric and physical‐field reconstruction. Many of the cases discussed in this paper are still more directly related to curvy surface measurement than to surface reconstruction.

**TABLE 1:** Critical factors in curvy surface reconstruction and their classification, advantages, limitations, related figures, and challenges.

	Factor		Classification	Advantages	Limitations	Applications	Figures	Challenges		
**Curvy Surface Reconstruction** (*General curvy surface reconstruction incorporates both geometric and physical dimensions, representing an upgrade over conventional geometric reconstruction* *that emphasizes solely spatial relationships*)	**Measurement** **methods** (*Obtaining* *precise* *discrete data*)	**Non‐contact** **measurement** **methods**	Optical measurements	High‐speed, Non‐invasive, Without media	Blind angles, High sensitivity, Restricted penetration depth	Face recognition, Wind tunnel test, Blood vessel monitoring	Figure 2 Figure 3	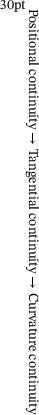	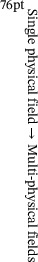	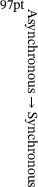
			Acoustic measurements	Deeper penetration	Lower spatial resolution, Media dependence	Visceral monitoring, Underwater detection	Figure 4			
			Photoacoustic measurements	Balanced high‐resolution and deep‐penetration	Light scattering, Stricter media dependence	Breast monitoring, Microvessels monitoring	Figure 5			
		**Contact** **measurement** **methods**	Sensitive coatings	Compatibility, Large‐area, Low‐consumption	Coating stability, Surface‐only applicability	Aerodynamic test, Environmental monitoring	Figure 6			
			Flexible electronics	Ultrathin, Real‐time & In‐situ, Continuous	Limited material selection, Mechanical fragility	Implantable sensing, Aerodynamic sensing, Electromagnetic cloaking	Figure 7 Figure 8			
			Flexible fibers	Mechanical adaptability, Seamless integration	Mechanical mismatch, Limited elasticity	Embedded/Multilayer sensing, Structural health monitoring	Figure 9 Figure 10			
	**Reconstruction algorithms** (*Building rational connection of discrete data*)	**Physical model‐based** **algorithms** (Ko method, FEM, iFEM)		Small database, Physical consistency	Sample shapes, No scalability	Structures with governing equations (e.g., rods, beams, plates, shells)	Figure 11			
		**Data training‐driven** **Algorithms** (Machine/Deep learning, Physics‐informed AI)		Scalable AI algorithms, Large datasets	Physical laws or multi‐field interactions not yet integrated	Applicability to arbitrary complex curvy surfaces	Figure 12			

## 2 Non‐Contact Measurements

Measurement methods for curvy surfaces can be broadly classified into non‐contact and contact categories, depending on whether the measuring system physically interacts with the targets. This section focuses on non‐contact measurement methods, while Section 3 introduces contact measurement methods. According to the underlying physical principles, non‐contact measurements can be further subdivided into optical [57, 58, 59, 60, 61], acoustic [62, 63, 64, 65, 66, 67, 68, 69], and photoacoustic measurements [70, 71, 72, 73, 74, 75, 76, 77], with optical measurements currently occupying a prominent position. Compared to contact counterparts, non‐contact methods have the advantages of preserving the structural integrity of the targets and minimizing physical interference during measurement or manipulation. These benefits are particularly critical in applications involving delicate or sensitive surfaces, where contact could cause damage, deformation, or contamination [135, 136, 137]. Furthermore, non‐contact methods typically support faster data acquisition over larger areas, enabling more efficient, high‐resolution assessments.

### 2.1 Optical Measurements

Optical measurements leverage the fundamental principles of light–matter interactions to determine the characteristic parameters or physical properties of a target. When a target is illuminated by a specific light source, its intrinsic optical properties, such as refractive index, absorption coefficient, surface roughness, and structural dimensions, modify the key attributes of the reflected or transmitted light, including intensity, phase, wavelength, polarization state, and propagation direction [138, 139]. By measuring and analyzing these modified optical signals, the corresponding physical quantities or structural characteristics can be indirectly inferred. Generally, optical measurements can be classified into two categories: passive [140, 141, 142, 143] and active [144, 145, 146, 147, 148], depending on the need for an external light source. Passive optical measurements rely on ambient or naturally occurring light to analyze the target [141, 142, 143], while active optical measurements require an external light source to illuminate the object and induce light–matter interactions for further analysis [146, 147, 148]. Optical measurements offer several significant advantages over other modalities, including the rapid signal propagation, non‐reliance on propagation media, and non‐contact operation. These features allow for versatile applications, enabling high‐precision, high‐speed, and noninvasive visualization and characterization of complex structures. Representative examples include healthcare and biomedical monitoring [45, 46, 47], wind tunnel tests of aircraft [48, 49, 50], and face recognition [149, 150, 151].

**FIGURE 2:**
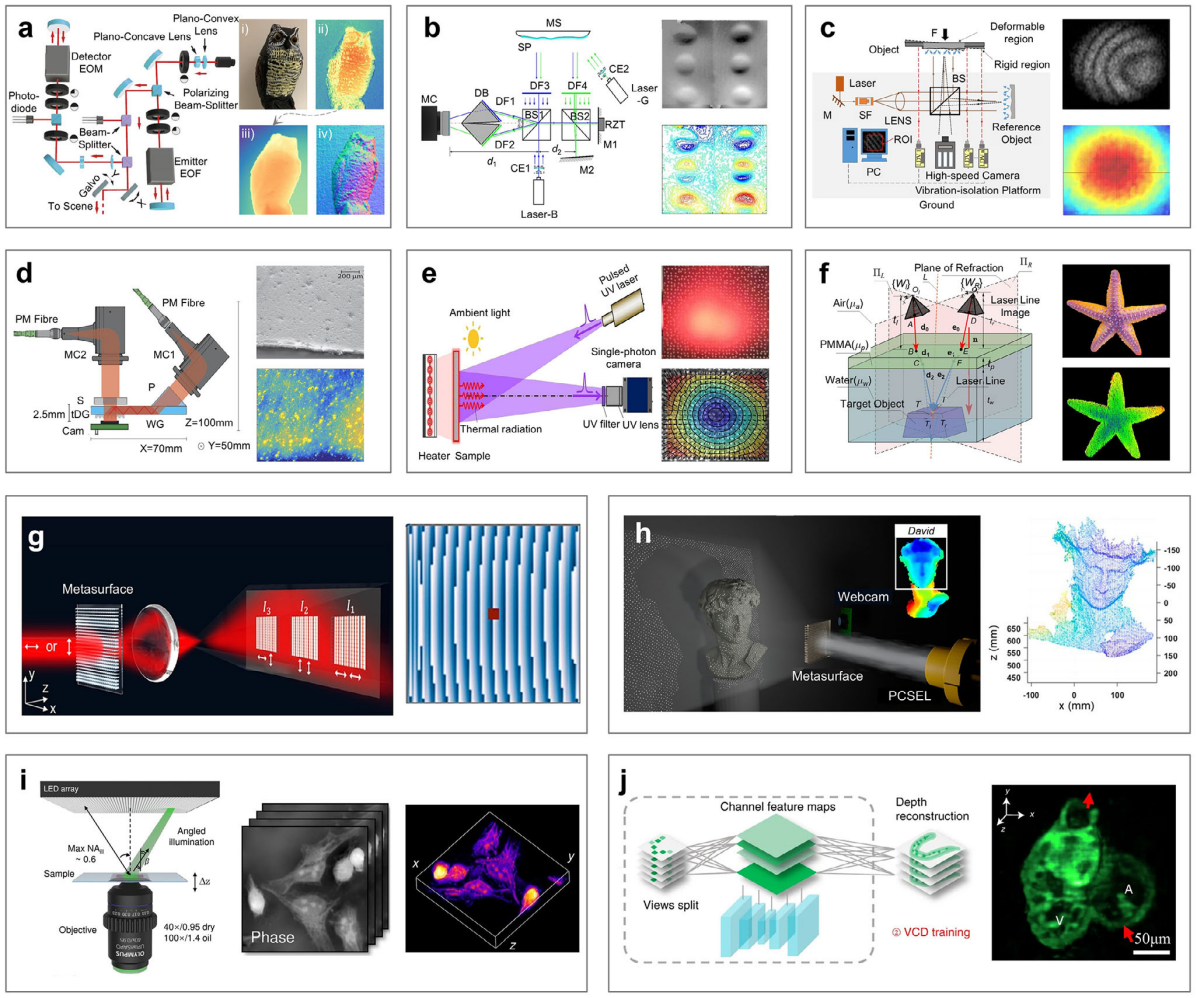
Optical measurement methods for the solid field. (a) All‐optical neural TOF imaging system for owl's depth imaging. (b) Dual‐channel speckle interferometry method for deformation measurements. (c) Electronic speckle pattern interferometry method for deformation measurements. (d) On‐chip digital holographic interferometry for wavefront deformation measurements of transparent samples. (e) Active‐imaging DIC method for thermal deformation measurements. (f) Binocular SLS with a laser for 3D underwater shape reconstruction. (g) Schematic diagram of the polarization multiplexing metasurface (left) and the phase distribution of the first diffraction order (right). (h) Metasurfaces and photonic crystal surface‐emitting lasers for 3D facial reconstruction of *David*. (i) Optical diffraction tomography for 3D biological cells imaging. (j) Light‐field microscopy and deep learning for 3D volumetric reconstruction of beating myocardium. (a) Reproduced with permission [144]. Copyright 2023, ACM. (b) Reproduced with permission [140]. Copyright 2023, Elsevier. (c) Reproduced with permission [141]. Copyright 2024, Optica. (d) Reproduced under the terms of the CC‐BY 4.0 [157]. Copyright 2023, Optica. (e) Reproduced with permission [146]. Copyright 2025, Elsevier. (f) Reproduced with permission [142]. Copyright 2023, IEEE. (g) Reproduced with permission [158]. Copyright 2024, American Chemical Society. (h) Reproduced with permission [150]. Copyright 2024, American Chemical Society. (i) Reproduced under the terms of the CC‐BY 4.0 [143]. Copyright 2022, Optica. (j) Reproduced with permission [159]. Copyright 2021, Springer Nature.

Optical measurements for the solid‐field applications are shown in Figure 2, where the commonly investigated physical quantities include geometric morphology, optical parameters, stress–strain distributions, and temperature fields. Representative optical measurements include Time‐of‐Flight (TOF) [152, 153], Electronic Speckle Pattern Interferometry [154], Digital Image Correlation (DIC) [155], Structured Light Scanning (SLS) [58], and Spectroscopic Imaging [156]. Early optical measurements typically employed a single camera for passive measurements. However, these early systems suffered from two major limitations: (i) a heavy dependence on ambient light, and (ii) limited depth perception. To address the first issue, an all‐optical neural TOF imaging system was developed, enabling gigahertz frequency structural imaging by measuring the round‐trip times of emitted photons (Figure 2a) [144]. As the accuracy of single‐light‐source methods proved insufficient, Yuan et al. introduced a dual‐channel speckle interferometry method, incorporating a dual biprism and dichroic filters to directly visualize surface deformations (Figure 2b) [140]. Nevertheless, noise interference remained a persistent challenge. To mitigate this, researchers integrated Laser Doppler Vibrometry (LDV) with speckle‐based measurements (Figure 2c) [141], thereby enabling dynamic deformation sensing under noisy conditions. To further expand capabilities, a digital holographic interferometry approach based on a Mach–Zehnder configuration was introduced (Figure 2d) [157], leveraging wavefront measurements to expand the field of view. Recently, to address interference and thermal constraints, a DIC system that directly inscribes speckle patterns was proposed (Figure 2e) [146], enabling thermal deformation measurements at elevated temperatures via combining ultraviolet bandpass filtering and advanced imaging.

To address the second issue of limited measurement depth, Ou et al. combined SLS with a laser source (Figure 2f) [142]. With binocular lenses for shape reconstruction and a laser for light‐field compensation, the system enabled 3D underwater imaging. However, reflection‐induced signal loss limited traditional SLS to static measurements on rough surfaces. In response, a polarized metasurface‐based SLS system was developed (Figure 2g) [158]. By dynamically controlling the polarization of incident light, this system enabled high‐frequency microscopic imaging. Building on this, researchers further integrated metasurfaces with photonic crystal surface‐emitting lasers, resulting in a low‐power, super‐resolution imaging system (Figure 2h) [150]. Recently, 3D microscopic imaging in biomedical applications has garnered substantial attention. To achieve high‐resolution tomographic imaging of cellular structures, Zhou et al. introduced an intensity‐transport method grounded in optical diffraction tomography (Figure 2i) [143]. Furthermore, the integration of emerging deep‐learning techniques with light‐field microscopy enabled high‐speed, high‐resolution cellular imaging, broadening the biomedical applicability of optical measurements (Figure 2j) [159].

**FIGURE 3:**
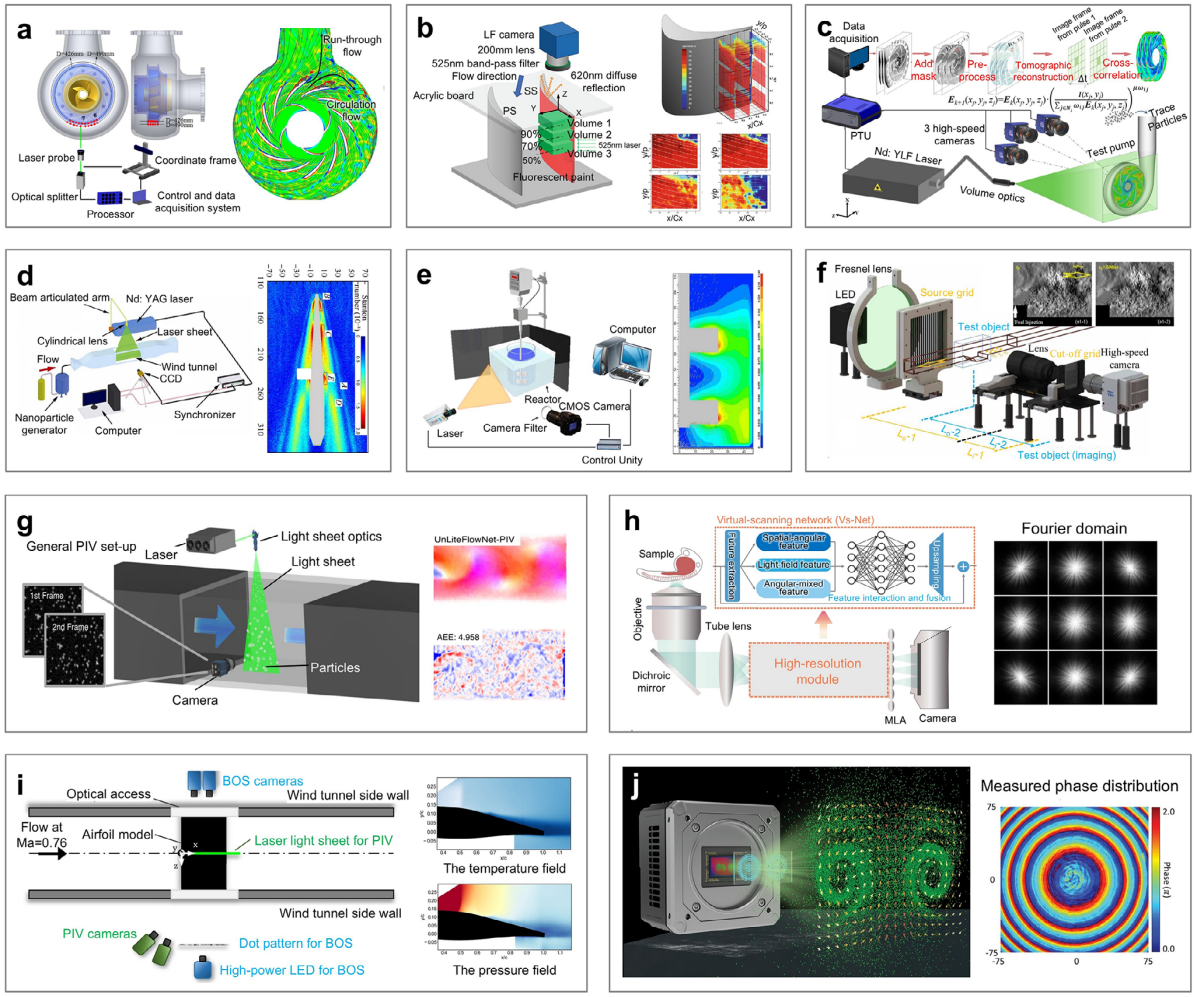
Optical measurement methods for the flow field. (a) LDV for measuring pressure pulsations and the flow field in a nuclear reactor coolant pump. (b) LF‐PIV method for high‐speed instantaneous streamwise velocity distribution imaging. (c) Tomo‐PIV measurement system. (d) NPLS system and heat flux distribution of a swept fin‐induced flow field. (e) LIF technique for flow field measurement in water. (f) An enhanced BOS method for scramjet combustor schlieren imaging. (g) Deep recurrent optical flow learning for flow‐field measurements. (h) Supervised‐learning network method for high‐resolution cell volumetric imaging. (i) Multi‐field synchronous measurements under transonic shock conditions based on PIV and BOS. (j) Meta‐Lens PIV for flow field measurements. (a) Reproduced with permission [145]. Copyright 2020, Elsevier. (b) Reproduced with permission [147]. Copyright 2022, Springer Nature. (c) Reproduced with permission [148]. Copyright 2024, AIP. (d) Reproduced under the terms of the CC‐BY‐NC 4.0 [168]. Copyright 2024, Springer Nature. (e) Reproduced with permission [169]. Copyright 2021, Elsevier. (f) Reproduced with permission [163]. Copyright 2024, IOP. (g) Reproduced with permission [170]. Copyright 2021, Springer Nature. (h) Reproduced with permission [171]. Copyright 2023, Springer Nature. (i) Reproduced under the terms of the CC‐BY 4.0 [172]. Copyright 2024, Springer Nature. (j) Reproduced with permission [173]. Copyright 2023, Wiley.

Beyond solid fields, optical measurements are also powerful tools for characterizing flow fields. These methods can be broadly classified into two types: particle‐tracking‐based methods [160, 161, 162] and optical‐principle‐based methods [163, 164, 165, 166]. Particle‐based methods include LDV [167], Particle Image Velocimetry (PIV) [160], Particle Tracking Velocimetry [59], Laser‐Induced Fluorescence (LIF) [161], and Nano‐tracer‐based Planar Laser Scattering (NPLS) [162]. In contrast, optical‐principle‐based methods include Background‐Oriented Schlieren (BOS) [164] and Light‐Field Microscopy [165]. LDV is commonly employed to measure pressure pulsations and flow characteristics in unsteady flow (Figure 3a) [145]. Recently, PIV and its derivatives have become popular for flow field measurement. For example, LF‐PIV enables 3D, high‐speed imaging with a single camera, reducing system complexity significantly (Figure 3b) [147]. Then Tomo‐PIV employs additional cameras and higher tracer particle density to achieve high spatiotemporal resolution in centrifugal pumps (Figure 3c) [148]. However, traditional PIV faces challenges in hypersonic flow environments, where large tracer particles and weak signal intensities hinder effective measurement. To address these limitations, NPLS was developed by Yi et al., employing nanoparticles to visualize transient flow fields and thermal‐flow density gradients (Figure 3d) [168]. Additionally, LIF uses laser excitation to elevate tracer particles within the flow to higher energy states (Figure 3e) [169]. When combined with PIV and computational fluid dynamics analyses, LIF can provide comprehensive insights into complex flow dynamics and scalar transport phenomena.

Among optical‐principle‐based methods, Zhao et al. enhanced the traditional BOS method by customizing the light source and exposure time, thereby reducing background light interference (Figure 3f) [163]. More recently, as the data volume generated by techniques such as PIV and Light‐Field Microscopy continues to expand, conventional computational models face increasing challenges related to resolution limitations and motion artifacts. To address these issues, advanced computational algorithms have been integrated to achieve ultrahigh‐accuracy flow‐field measurements (Figure 3g) [170] and ultrafast 3D imaging (Figure 3h) [171]. Furthermore, researchers achieved multi‐field synchronous measurements under transonic shock conditions by combining PIV with BOS, enabling the simultaneous characterization of density gradients, pressure distributions, and temperature fields around aircraft wings (Figure 3i) [172]. Finally, the advent of binocular metalenses, composed of artificial nano‐array antennas, offered a new avenue for miniaturization and low‐power consumption in PIV‐based measurements (Figure 3j) [173]. This innovation is particularly beneficial in space‐constrained environments.

Optical measurement methods are among the most widely employed non‐contact methods, offering rapid signal transmission and micro‐ to nanoscale accuracy and resolution. They are applicable to both solid and flow field measurements. Nonetheless, several inherent limitations warrant attention: (i) Dependence on Sample Surface Conditions: Excessive roughness, contamination, and specialized surface textures may compromise the stability and repeatability of optical signals. (ii) High Sensitivity to Environment: Vibration, air turbulence, ambient light interference, and other environmental matters can present significant challenges. (iii) Restricted Penetration Depth: When thicker structures are irradiated with light, photons are absorbed and scattered by scanning media, with a penetration depth that depends on the wavelength. (iv) Inability to Move Measurements and Blind Angles: The fixed camera greatly affects its application scenarios. In summary, future developments in optical measurement methods may focus on: (a) exploring ultrafast laser technologies to enhance speed, resolution, and temporal accuracy, (b) developing innovative optical components, such as metamaterial imaging and metasurface holographic elements, and (c) integrating optical methods with complementary modalities to enable multimodal characterization of structures.

### 2.2 Acoustic Measurements

**FIGURE 4:**
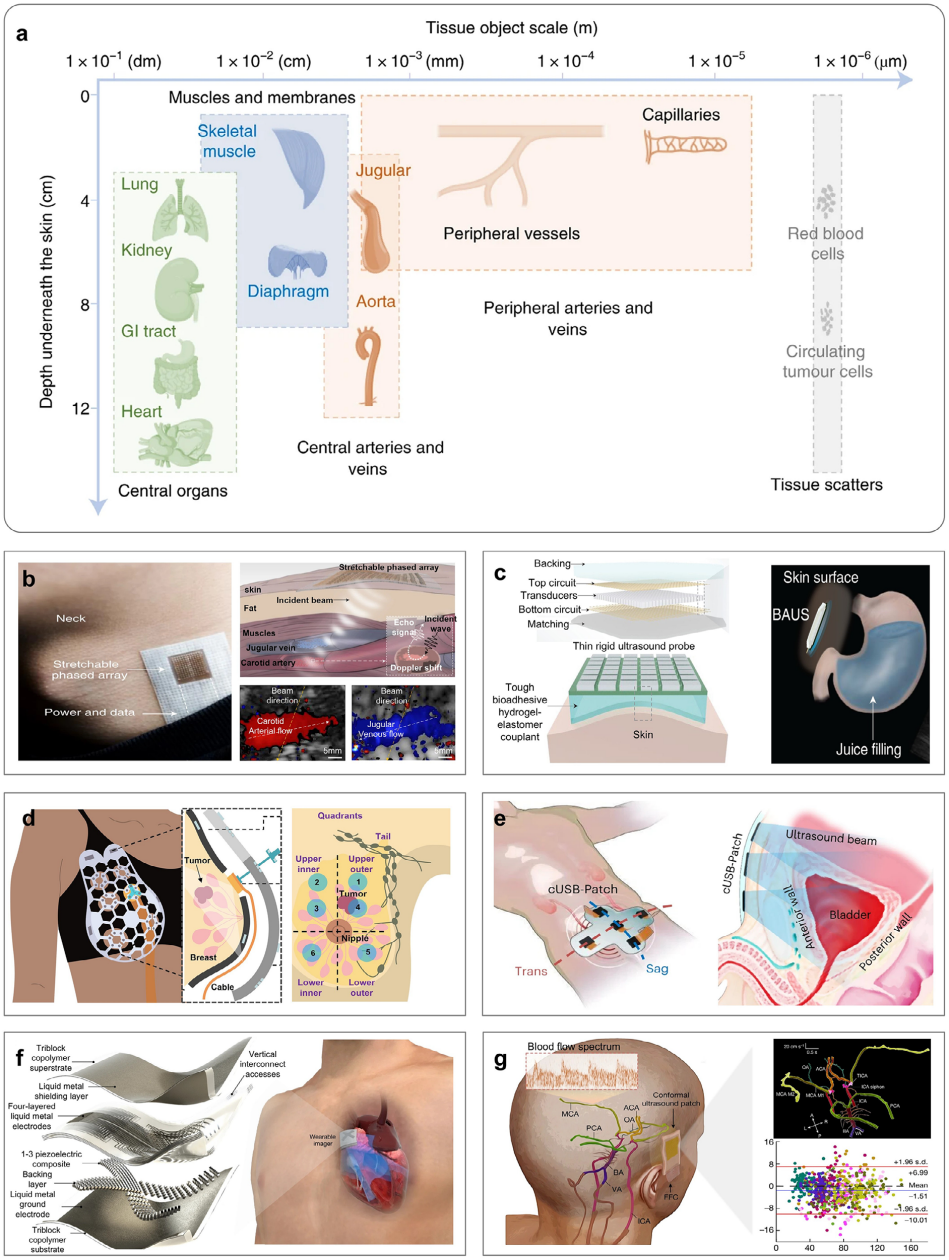
Acoustic measurement methods. (a) The depth and size of several representative human tissues. (b) A wearable ultrasonic phased‐array device for monitoring hemodynamic signals. (c) A bioadhesive ultrasound device for continuous imaging of the stomach. (d) Ultrasonic device for measuring smaller breast tissue and cysts. (e) Ultrasonic patch for bladder‐volume measurement. (f) Ultrasonic patches for measuring left‐ventricle volume and monitoring cardiac performance indicators. (g) Conformal ultrasound patches for blood‐flow imaging in complex cerebral vessels. (a, b) Reproduced with permission [63]. Copyright 2021, Springer Nature. (c) Reproduced with permission [64]. Copyright 2022, AAAS. (d) Reproduced with permission [184]. Copyright 2023, AAAS. (e) Reproduced with permission [185]. Copyright 2023, Springer Nature. (f) Reproduced under the terms of the CC‐BY 4.0 [65]. Copyright 2023, Springer Nature. (g) Reproduced with permission [66]. Copyright 2024, Springer Nature.

Acoustic measurement utilizes the propagation behavior of sound waves in a medium to obtain the physical parameters and characteristics of targets. As sound waves travel through a medium, their interactions, such as reflection, refraction, scattering, and diffraction, are influenced by the medium's density, elastic modulus, and absorption coefficient. These interactions result in changes to the wave's amplitude, phase, frequency, and velocity [174, 175]. The modified acoustic signals are then converted into electrical signals, which can be analyzed to determine key properties of the target, including thickness, defect locations, elastic modulus, and density [176]. Compared with optical measurements, acoustic measurements offer greater penetration depth. For example, as illustrated in Figure 4a [63], in biomedical monitoring, optical measurements are typically limited to imaging peripheral blood vessels at depths of less than 6 cm. In contrast, acoustic measurements can penetrate tissues beyond 12 cm, enabling the examination of decimeter‐scale internal structures such as the heart and major arteries. At present, acoustic measurements are widely applied in various domains, including industrial nondestructive testing [177, 178, 179], organ and cellular imaging [64, 65, 66], and underwater communication [180, 181, 182], owing to their deep penetration, noninvasive nature, and robust performance in turbid environments. Acoustic measurements for curvy surface reconstruction are exemplified by prenatal ultrasound imaging and underwater sonar detection [183]. In prenatal ultrasound examinations, repeated data acquisition from multiple angles using an ultrasound probe can be fused through imaging algorithms to reconstruct and display the baby's contour in real time, even resolving facial expressions and limb details. Similarly, underwater sonar detection employs acoustic beam scanning and integrates echoes from multiple angles to reconstruct both the shape and motion information of dynamic objects.

Ultrasonic measurement is a cutting‐edge technique in acoustic measurements, which has been widely used in medical monitoring applications (Figure 4). For instance, Wang et al. introduced a wearable ultrasonic phased‐array device designed to monitor hemodynamic signals in tissues located up to 14cm beneath the skin (Figure 4b) [63]. Moreover, this phased array beam set a precedent for large‐area wearable diagnostics. Subsequently, the same team first came up with a thinner and softer bioadhesive ultrasound device, capable of continuously imaging various internal organs over a 48‐h period (Figure 4c) [64]. This advancement significantly improved comfort and imaging continuity for long‐term physiological monitoring. To enable better conformability to the human body, particularly for soft tissue tracking, Du et al. pioneered an ultrasonic measurement method that better conforms to curvy anatomical surfaces (Figure 4d) [184]. Inspired by natural geometries, this device provided an expanded field of view, making it especially useful for breast tissue imaging. Additionally, a conformable transducer was invented to enhance the intensity of the transmitted signal. When integrated with ceramic materials, this configuration enabled higher‐frequency ultrasonic imaging for bladder volume measurement, which is a feat not achievable with traditional hydrogels and piezoelectric crystals (Figure 4e) [185]. Recently, the incorporation of deep learning into ultrasonic systems has further expanded their potential [186]. For example, Hu et al. developed ultrasonic patches capable of continuous left‐ventricle volume measurement and real‐time monitoring of cardiac performance (Figure 4f) [65]. This integration compensated for motion artifacts, such as those caused by deep breathing post‐exercise, and supported real‐time physiological monitoring. Growing clinical interest is now focused on neurological applications, particularly in blood‐flow imaging of cerebral vessels [187]. In this context, Zhou et al. proposed the first conformal ultrasound patches for 3D blood‐flow imaging in microtissues, offering a powerful platform for both clinical diagnostics and fundamental hemodynamic research (Figure 4g) [66]. Crucially, flexible ultrasound patches are susceptible to bending or stretching, which alters the relative positions and orientations of array elements and consequently degrades spatial resolution and introduces localization errors. There are two complementary strategies commonly adopted: structural design and algorithm compensation. Structural design mitigates or eliminates relative displacement among elements through mechanical or materials design, such as hydrogel. Algorithmic compensation corrects measurements by sensing or estimating the post‐deformation element geometry and incorporating it into the reconstruction model.

Acoustic measurement methods are well‐suited for penetrating various media to obtain internal information about target structures. However, there still exist several limitations: (i) Trade‐off between penetration and resolution: With the increase of sound frequency, the wavelength will become shorter, and the penetration depth will be reduced. (ii) Resolution Loss at High Imaging Speeds: High‐speed imaging requires higher pulse frequencies, reducing signal strength and increasing echo interference, thus lowering resolution. (iii) Frequent manual relocation requirements: Acoustic energy is primarily concentrated along the central axis, while significant attenuation occurs at the edges. Therefore, even slight displacement of the deep targets may cause the device to lose focus, which requires frequent manual relocation. (iv) Sensitive to dynamic measuring: Noise in dynamic scenes affects the phase and frequency of sound waves and overlaps with echo signals, misleading measurement results. Looking ahead, future developments in acoustic measurements will likely focus on miniaturization, integration, and customization. Specific directions include: (a) Developing novel transducers based on acoustic metamaterials or metasurfaces, (b) Integrating AI algorithms for measuring complex, flexible surfaces, (c) Advancing wireless energy transmission technologies to support untethered systems, (d) Combining with optical or other modalities for multimodal imaging, and (e) Developing fully integrated devices with soft front‐end electronics for conformable, wearable applications.

### 2.3 Photoacoustic Measurements

Photoacoustic measurement is a hybrid technique that exploits the acoustic response generated by light–matter interactions to reveal internal structural or material characteristics of a target. By combining the high temporal and spatial resolution of optical measurements with the deep penetration capabilities of acoustic methods, photoacoustic imaging offers a powerful, noninvasive diagnostic approach. In a typical setup, a pulsed light source irradiates the target, allowing it to absorb light energy over a very short duration (on the order of microseconds to nanoseconds). This absorbed energy is rapidly converted into heat, inducing localized thermal expansion or phase transitions, which in turn generate ultrasonic waves within the target medium [188]. By detecting and analyzing the arrival time, amplitude, and frequency spectrum of the emitted ultrasound, researchers can reconstruct the light absorption distribution and infer internal structural or material properties. Additionally, photoacoustic measurements support molecular imaging and multi‐channel detection, capitalizing on the target's spectrally dependent absorption characteristics across different wavelength ranges [189]. This feature allows for functional and compositional analysis at both macroscopic and microscopic levels. Notably, both acoustic and photoacoustic measurement methods require a coupling medium, although their property requirements differ. For acoustic measurements, the medium primarily needs to ensure effective acoustic coupling, emphasizing acoustic‐impedance matching, low attenuation, absence of bubbles, and stable adhesion, while optical transparency is generally nonessential. In contrast, photoacoustic measurements demand both optical and acoustic transmission. The coupling medium must allow efficient delivery of excitation light to the sample with minimal scattering or absorption, while simultaneously enabling the generated acoustic waves to return to the transducer with low loss. Photoacoustic techniques have found widespread application in medical laboratories [72, 73, 74], computed tomography [190, 191, 192, 193], and microparticle manipulation and control [194, 195, 196], among others, making them increasingly valuable in both clinical and research settings.

**FIGURE 5:**
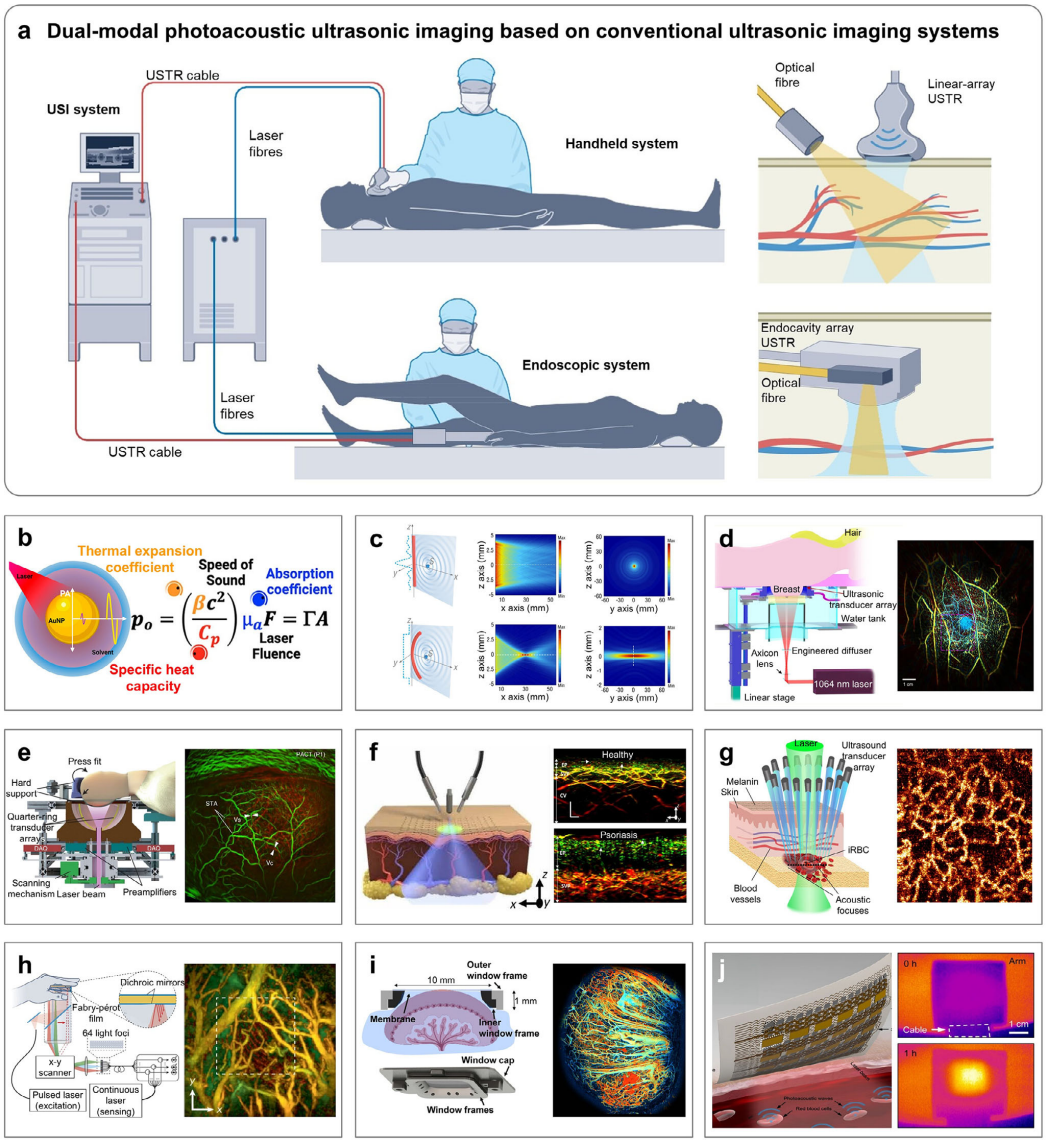
Photoacoustic measurement methods. (a) Dual‐modal photoacoustic imaging based on ultrasonic imaging. (b) Schematic diagram of photoacoustic measurements. (c) Schematic diagram for different bending degrees of optical fiber under a point ultrasonic source. (d) PACT imaging for human breast. (e) PACT for 3D imaging of the human brain. (f) A second‐generation ultrawideband photoacoustic system for microvessel imaging. (g) A photoacoustic‐based cell analyzer for monitoring red blood cells. (h) PACT dynamic imaging of microvessels. (i) Photoacoustic imaging for in vivo placental imaging. (j) Flexible electronic patch for photoacoustic imaging. (a) Reproduced with permission [73]. Copyright 2024, Springer Nature. (b) Reproduced with permission [197]. Copyright 2020, American Chemical Society. (c) Reproduced under the terms of the CC‐BY 4.0 [198]. Copyright 2019, Optica. (d) Reproduced under the terms of the CC‐BY 4.0 [190]. Copyright 2018, Springer Nature. (e) Reproduced with permission [191]. Copyright 2021, Springer Nature. (f) Reproduced with permission [192]. Copyright 2022, AAAS. (g) Reproduced under the terms of the CC‐BY‐NC 4.0 [193]. Copyright 2024, Springer Nature. (h) Reproduced with permission [76]. Copyright 2024, Springer Nature. (i) Reproduced with permission [199]. Copyright 2024, AAAS. (j) Reproduced under the terms of the CC‐BY 4.0 [200]. Copyright 2022, Springer Nature.

To date, photoacoustic measurements have been widely used to monitor organs and blood vessels in various medical imaging applications. Early implementations relied on bulky setups or hand‐held probes, which typically integrated a short‐pulse light source and an ultrasonic transducer into a portable system. In these configurations, laser irradiation (illustrated as the yellow shadow) induced localized thermal expansion, generating photoacoustic signals, which were subsequently detected by the ultrasonic transducer (blue shadow). These signals were then processed into visual images to enable the identification and differentiation of tissue types (Figure 5a) [73]. The wavelength of the light source plays a critical role in determining imaging specificity. For instance, ultraviolet light is effective for imaging DNA and proteins, whereas visible to near‐infrared region‐I (NIR‐I) wavelengths are suited for visualizing vascular morphology and molecular concentrations. Meanwhile, near‐infrared region‐II (NIR‐II) wavelengths are advantageous for imaging collagen and lipid structures [74]. A schematic of these wavelength‐dependent imaging principles is provided in Figure 5b [197]. In addition to the light source's frequency, the geometry of the light path significantly influenced imaging performance. Figure 5c presents schematic diagrams comparing a linear optical fiber sensor and an optical fiber sensor with a 3 cm bending radius under a point ultrasonic source [198]. These diagrams demonstrate how the spatial sensitivity distribution can be adjusted to enhance or target specific ultrasonic signal responses, thereby improving image resolution and measurement precision.

Impressively, Lin et al. designed a single‐breath‐hold photoacoustic computed tomography (PACT) capable of visualizing complex angiographic structures in the human breast during respiration (Figure 5d) [190]. For decades, the limited spatial resolution and slow response times of conventional PACT systems have posed significant challenges for 3D brain imaging. To address this, Na et al. successfully achieved the first in vivo human brain imaging using photoacoustic imaging by deploying a large‐scale ultrasonic transducer array (Figure 5e) [191]. To further advance clinical applications, Hindelang et al. developed a second‐generation ultrawideband photoacoustic mesoscopic system for skin disease assessment, enabling detailed imaging of microvasculature and inflammatory features (Figure 5f) [192]. Unlike traditional methods that rely on visual inspection and palpation, photoacoustic imaging offers a quantitative and high‐resolution approach for dermatological diagnostics. Photoacoustic techniques have also been extended to cell‐detection platforms. For instance, Yadem et al. introduced a photoacoustic‐based cell analyzer capable of monitoring red blood cells infected with malaria, demonstrating its utility for point‐of‐care diagnostics (Figure 5g) [193]. With the progression of photoacoustic technology, researchers have turned their attention to microscale structures. A Fabry–Pérot scanner based on PACT enables dynamic imaging of microvessels, offering a new pathway for diagnosing and treating microvascular lesions (Figure 5h) [76]. Beyond blood vessels and tissues, photoacoustic imaging has been applied to in vivo placental imaging, expanding its relevance to maternal‐fetal health (Figure 5i) [199]. However, most existing photoacoustic methods still rely on expensive equipment or radioactive tracers and are incapable of continuous monitoring [75]. In response, Gao et al. pioneered the integration of ultrasonic transducers and laser emitters into flexible electronic patches, significantly enhancing the potential of wearable photoacoustic systems (Figure 5j) [200]. This innovation represents a major step forward in enabling real‐time, noninvasive monitoring and opens new frontiers for personalized healthcare applications.

Currently, photoacoustic measurements have enabled high‐resolution imaging of internal organs and tissues in the medical field, yet some challenges remain. (i) Light scattering: The uneven distribution of light within biological tissues limits imaging accuracy. Achieving precise measurement often requires repeated adjustments to illumination parameters. Moreover, scattering significantly restricts the maximum penetration depth of the light source. (ii) Manual Operation Sensitivity: The need for manual positioning of the device to achieve optimal imaging angles introduces variability. Even slight tilting or surface pressure can cause misalignment and degrade image quality. (iii) Limited Standardization and Reproducibility. Variations in device design, experimental conditions, and operator technique can result in inconsistent measurements, making it difficult to standardize protocols or ensure cross‐platform compatibility. Therefore, future photoacoustic measurements will likely emphasize real‐time capability, cost‐effectiveness, and portability. Potential development directions include: (a) Designing flexible ultrasonic transducers based on metasurfaces to enhance bandwidth and sensitivity. (b) Introducing novel nanomaterials as contrast agents to improve light absorption, stability, and biocompatibility. (c) Incorporating AI algorithms to improve imaging resolution and correct for motion‐ or respiration‐induced artifacts. (d) Integrating photoacoustic systems with complementary techniques such as infrared thermal imaging or speckle interferometry enables multi‐angle, multimodal detection and enhanced structural characterization.

## 3 Contact Measurements

Contact measurements are another important branch for measuring curvy surfaces. According to the perception mechanism of signals, contact measurements can be mainly divided into three categories: (i) sensitive coatings [78, 79, 80, 81, 82], (ii) flexible electronics [83, 84, 85, 86, 87], and (iii) flexible fibers [88, 89, 90, 91, 92]. Unlike indirect non‐contact measurements, which first convert the measured signal into optical or acoustic signals and then derive physical quantities, contact measurements obtain the mechanical response of targets through direct mechanical coupling. They have the advantages of being unaffected by environmental interference, enabling in situ and real‐time measurements, and providing higher accuracy. These methods are particularly useful in wearable healthcare [45, 46, 47], robot intelligent perception [201, 202, 203], flexible smart skin [204, 205, 206], etc.

### 3.1 Sensitive Coatings

Sensitive coatings are functional materials applied directly to the surface of a target, designed to generate detectable physical or chemical responses to variations in specific external stimuli. When exposed to changes in pressure, temperature, magnetic fields, strain, or chemical adsorption, the microstructure and state of the coating materials undergo alterations. These microscopic transformations lead to measurable changes in macroscopic physical properties such as optical, electrical, magnetic, or mechanical characteristics, which can then be detected and analyzed [79]. Compared with other contact measurements, sensitive coatings outperform in large‐area, continuously distributed measurements and low power consumption. Moreover, their thinness and superior surface conformability make them particularly well‐suited for integration with complex curvy geometries, ensuring high compatibility and mechanical adaptability [207]. Due to the underlying measurement mechanism involving collisions or energy transitions between functional materials and oxygen or surrounding media, the measured information is closer to the fluid dynamics or thermodynamic environment. Therefore, sensitive coatings are especially skilled in measuring and visualizing phenomena such as vortex structures (eddies) [121, 122, 123], boundary layer distributions [124, 125, 126], and heat transfer processes [208, 209, 210]. Nowadays, sensitive coatings are widely employed in various engineering applications, including aerospace wind tunnel measurements [209, 210], vehicle aerodynamic research [211, 212], and surface pressure monitoring of mechanical components [81, 82], where both high resolution and surface adaptability are critical.

**FIGURE 6:**
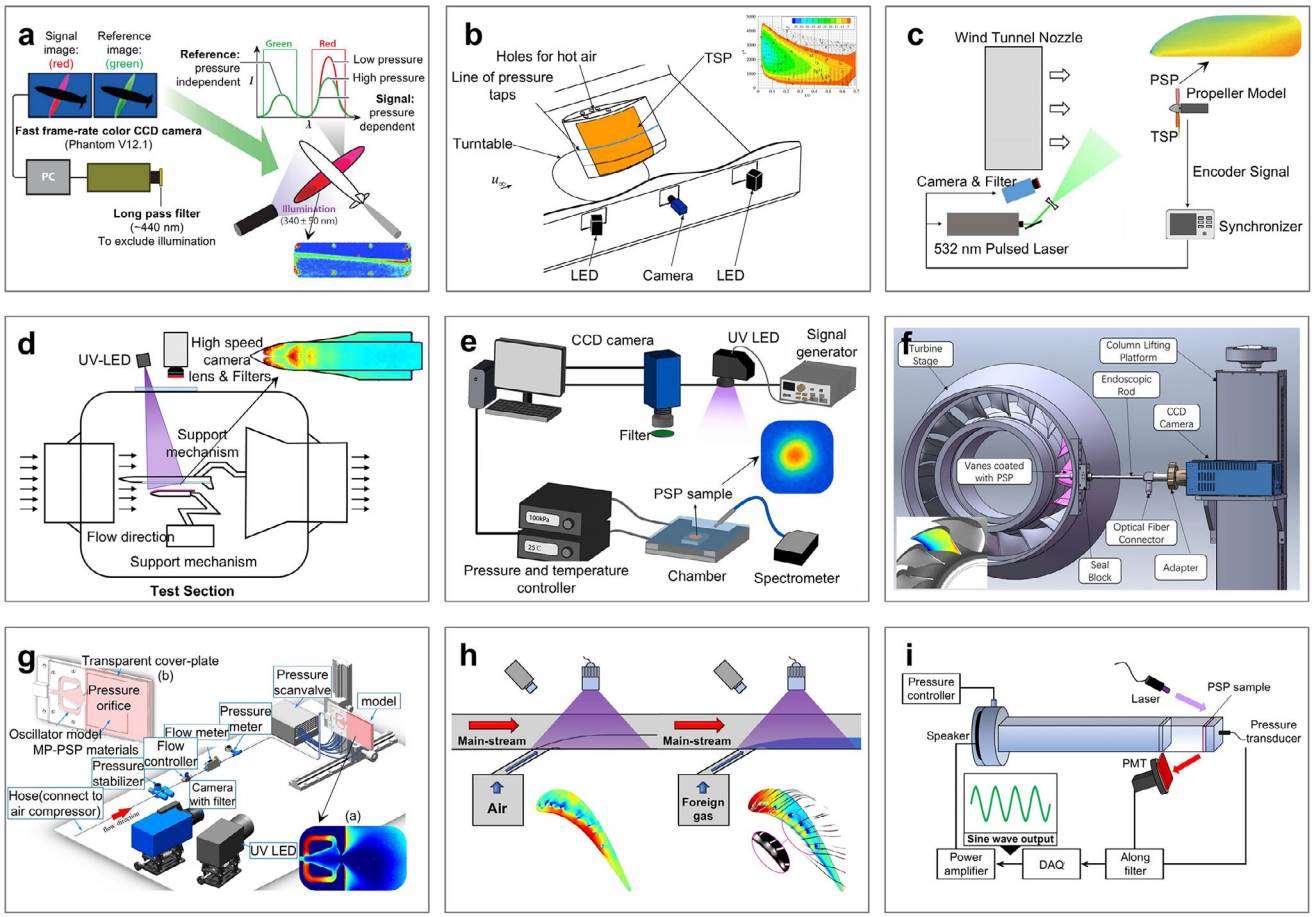
Sensitive coatings measurement methods. (a) Schematic diagram of PSP for capturing wing motion. (b) TSP visualization of the flow field over unpolished rear wing wind tunnel models. (c) PSP for pressure distribution measurements of rapidly rotating blades. (d) Temperature correction on PSP data based on TSP results. (e) New PSP measurement for temperature compensation in high‐speed aerodynamic testing. (f) An endoscopic PSP measurement system for reconstructing the 3D pressure field of engine blades. (g) PSP measurement for pressure fields of fluidic oscillators. (h) PSP calibration method based on mixed oxygen. (i) PSP with high emission intensity for unsteady pressure measurement. (a) Reproduced with permission [78]. Copyright 2014, Annual Review. (b) Reproduced with permission [209]. Copyright 2019, Elsevier. (c) Reproduced with permission [80]. Copyright 2022, Springer Nature. (d) Reproduced with permission [210]. Copyright 2020, Springer Nature. (e) Reproduced with permission [214]. Copyright 2022, Elsevier. (f) Reproduced with permission [81]. Copyright 2023, Springer Nature. (g) Reproduced with permission [82]. Copyright 2024, Springer Nature. (h) Reproduced with permission [123]. Copyright 2024, Elsevier. (i) Reproduced under the terms of the CC‐BY 4.0 [215]. Copyright 2024, IOP.

Pressure‐sensitive paint (PSP) and temperature‐sensitive paint (TSP) are the two most commonly used sensitive coatings. When a light source illuminates the surface, the luminescent materials in the paint are excited and subsequently emit fluorescence. Changes in pressure or temperature influence the energy levels of these luminescent molecules, thereby altering the intensity of fluorescence emission. This property allows for precise visualization and quantification of surface pressure and temperature distributions [79]. PSP and TSP have been extensively applied to measure both steady and unsteady flows in high‐speed and hypersonic regimes, such as surface pressure distributions on aerodynamic models [209, 210], and shock wave‐boundary layer interactions [124, 125, 126]. These methods were widely used in aerospace research and engine turbines (Figure 6). Figure 6a presents a schematic of the PSP‐based motion capture method for a wing model [78]. In this system, two optical filters, green and red channels, were employed to collect emission intensities. Their intensity ratio served to correct errors introduced by model motion, improving measurement stability. Additionally, TSP has also been shown to effectively visualize flow fields in wind tunnel models with unpolished surfaces, such as rear wings (Figure 6b) [209], demonstrating that its temperature sensitivity and spatial resolution were unaffected by surface roughness. Beyond static components, PSP is also used for measuring the pressure distribution on rapidly rotating blades, with its experimental results in good agreement with both force balance measurements and computational fluid dynamics simulations (Figure 6c) [80]. However, temperature‐induced errors remain one critical challenge in PSP, particularly under ultrahigh‐speed conditions [213]. To address this, Peng et al. developed a semi‐transparent coating that enhanced the illumination and imaging functions of the optical path and performed temperature correction on PSP data based on TSP results (Figure 6d) [210]. This approach successfully visualized orbiter flow fields at Mach 6. Furthermore, Gu et al. introduced a novel PSP formulation incorporating two luminescent groups with opposite temperature sensitivities, enabling built‐in temperature compensation in high‐speed aerodynamic testing environments (Figure 6e) [214].

With the continued advancement of PSP/TSP measurements, research efforts have increasingly turned toward applications involving complex structures. Traditional PSP/TSP techniques frequently face challenges in confined or obstructed environments, such as those found in engine blades, where the complex geometry limits direct optical access. To address this, Dong et al. developed an endoscopic PSP measurement system combined with a dynamic distortion correction technique, enabling accurate reconstruction of the 3D pressure field within enclosed blade structures (Figure 6f) [81]. Beyond enclosed surfaces like wings and blades, PSP has also been applied to study the internal and external pressure fields of porous structures. A representative example includes its use in analyzing fluidic oscillators (Figure 6g) [82]. Recent research has also focused on capturing air density variations and 3D unsteady flow fields. In high‐speed or complex flow environments, pressure distribution can be significantly influenced by density differences between ambient air and injected gases. To improve accuracy in such conditions, Tao et al. developed a mixed‐oxygen‐based calibration method, which effectively reduced pressure measurement deviations across a wide range of scenarios (Figure 6h) [123]. Moreover, for capturing unsteady pressure distributions under high‐speed sampling conditions, Kasai et al. proposed the concept of a pressure sensitivity coefficient (Figure 6i) [215]. By evaluating and selecting PSP materials with high emission intensity under elevated pressure, they identified optimal formulations capable of delivering accurate, time‐resolved measurements in rapidly fluctuating aerodynamic environments.

As a widely adopted method for surface measurements, sensitive coatings have achieved 3D reconstructions of pressure or temperature fields in various industrial settings, but some challenges remain: (i) High Measurement Requirements: Accurate PSP/TSP measurements depend heavily on careful excitation and detection light path design. Factors such as camera angles, shadow effects, and lighting uniformity must be meticulously managed to avoid blind spots and color deviations, particularly in cases involving large deformations. (ii) Poor Coating Stability: Under high‐speed flow or elevated temperature conditions, the coatings are susceptible to peeling, corrosion, or aging, which can lead to signal degradation and measurement drift over time. (iii) Inability to Probe Internal Structures: Due to their surface‐luminescence mechanism, sensitive coatings primarily capture information from surface interactions or very shallow regions of gas molecule collisions. This limits their ability to access subsurface or multilayer structural parameters. Looking forward, future development efforts are expected to focus on two key areas: (a) Advanced luminescent material design and coating process optimization. Enhancing the thermal, mechanical, and chemical stability of coatings will improve their performance in extreme environments such as high‐speed flows and high‐temperature conditions. At the same time, increasing the sensitivity of fluorescence lifetime or emission intensity to pressure and temperature variations will broaden the applicability of these materials. (b) Multimodal measurement integration: Coupling PSP/TSP methods with complementary techniques such as PIV and infrared thermography may enable synchronous measurement of multiple physical quantities, providing a more comprehensive understanding of complex flow and thermal phenomena.

### 3.2 Flexible Electronics

Flexible electronics are a class of electronic devices that can be stretched, bent, twisted, and deformed into arbitrary shapes, overcoming the limitations of traditional rigid integrated circuit technologies. As a promising next‐generation electronics platform, flexible electronics have broken through the longstanding bottlenecks and monopolies of conventional silicon‐ and metal‐based systems [216]. Since their introduction approximately two decades ago, they have attracted increasing attention due to both their intriguing physical properties and broad application potential. Unlike rigid electronics governed by Moore's law, flexible electronics are thin, lightweight, low‐modulus, and mechanically stretchable, making them effectively “invisible” when applied to surfaces with complex or dynamic geometries. These characteristics make them particularly well‐suited for measuring and visualizing complex, flexible, and time‐varying surfaces [217]. When subjected to mechanical strain, temperature fluctuations, or other physical stimuli, the resistance, capacitance, inductance, and electromagnetic field parameters within flexible electronic systems change accordingly. By analyzing these circuit‐level variations, it becomes possible to achieve real‐time, dynamic monitoring of the underlying surface or structural behavior. Flexible electronics provide an almost ideal platform for noninvasive, continuous, in situ, and real‐time measurement and 3D reconstruction of curvy surfaces, while maintaining comfort and conformability. Compared with other measurement techniques, flexible electronics also offer distinct advantages in integrability, programmability, and repeatability [115, 116, 117, 118, 119, 120]. To date, flexible electronics have demonstrated a wide range of novel applications. Representative examples included epidermal, implantable, and wearable electronics for human healthcare monitoring [218, 219, 220], artificial skin for robotics and human–robot interaction interfaces [201, 202, 203], and smart sensing skin for aerospace vehicles and structural health monitoring [204, 205, 206].

**FIGURE 7:**
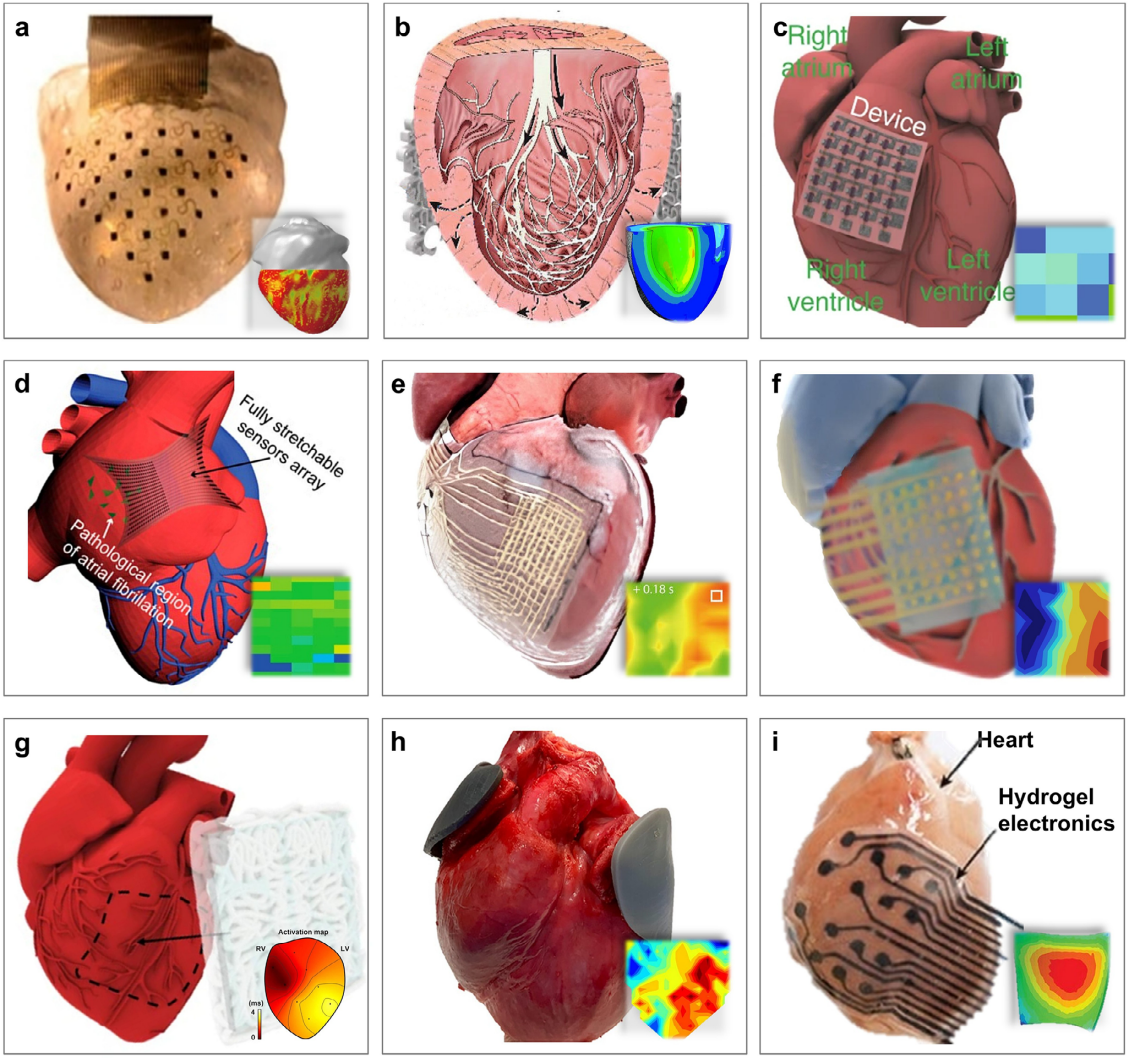
Flexible electronics for cardiac healthcare monitoring. (a) Conformal 3D elastic membrane for cardiac monitoring of temperature, deformation, and pH levels. (b) A low elastic modulus myocardial flexible mesh for recording electrocardiograms. (c) Flexible epicardial bioelectronics for temperature and strain sensing. (d) Flexible electrode array for high‐resolution mapping. (e) Flexible electronic sensing system in a humid environment. (f) Flexible electronics for stable imaging of mouse hearts. (g) Flexible bio‐patch for precise cardiac ECG monitoring. (h) Flexible electrode for 3D imaging and localization of the ventricle. (i) A 3D‐printed hydrogel‐based flexible sensor for long‐term, high‐precision epicardial physiological monitoring. (a) Reproduced under the terms of the CC‐BY 4.0 [221]. Copyright 2014, Springer Nature. (b) Reproduced with permission [222]. Copyright 2016, AAAS. (c) Reproduced with permission [223]. Copyright 2020, Springer Nature. (d) Reproduced with permission [224]. Copyright 2020, National Academy of Science. (e) Reproduced with permission [225]. Copyright 2022, AAAS. (f) Reproduced with permission [226]. Copyright 2021, Springer Nature. (g) Reproduced with permission [227]. Copyright 2023, Springer Nature. (h) Reproduced under the terms of the CC‐BY 4.0 [228]. Copyright 2023, Wiley. (i) Reproduced with permission [229]. Copyright 2023, Wiley.

Here, we highlight two of the most representative application scenarios, the heart and aircraft, to demonstrate the potential of flexible electronics. First, Figure 7 showcases their role in implantable electronics for the measurement and 3D reconstruction of cardiac function. By adhering directly to the epicardium, flexible electronics enable precise and real‐time monitoring of critical cardiac parameters, such as heart rhythm, ischemic heart disease assessment, and epicardial substrate mapping. For instance, Xu et al. pioneered the use of 3D printing to fabricate an elastic membrane that conforms precisely to the heart's surface (Figure 7a) [221], enabling the monitoring of temperature, deformation, and pH throughout the cardiac cycle. Similarly, Park et al. introduced a low‐modulus myocardial wrapping structure that maintained conformal contact during heart motion and integrated a mesh electrode array for electrocardiogram recording (Figure 7b) [222]. With continued progress in flexible manufacturing, attention has shifted to miniaturized, portable, and easily fabricated patches. For example, the epicardial bioelectronic patch shown in Figure 7c enabled temperature and strain sensing during physiological activity and incorporated therapeutic functions such as electrical pacing and thermal ablation [223]. However, current devices still face challenges related to low spatial resolution and electromechanical decoupling during dynamic heart motion. In response, Liu et al. developed a high‐density, fully elastic electrode array capable of stable in vivo mapping while preserving both mechanical compliance and electrochemical performance (Figure 7d) [224]. To better replicate the humid cardiac environment, Hwang et al. designed a flexible electronic system capable of stable operation under moisture exposure (Figure 7e) [225]. Building on this, stable monitoring of mouse hearts has been successfully achieved (Figure 7f) [226]. However, issues such as long‐term adhesion and fatigue‐induced fractures persist. To overcome these, Choi et al. introduced a bioelectronic patch that adhered instantly to heart tissue and retained mechanical integrity after thousands of tensile loading cycles (Figure 7g) [227]. Additionally, the flexible electrodes shown in Figure 7h achieved 3D imaging and localization of the ventricle [228]. To overcome limitations in the processability of traditional hydrogel‐based electronics, Wang et al. invented a 3D‐printed hydrogel with high mechanical flexibility and shear strength (Figure 7i) [229].

**FIGURE 8:**
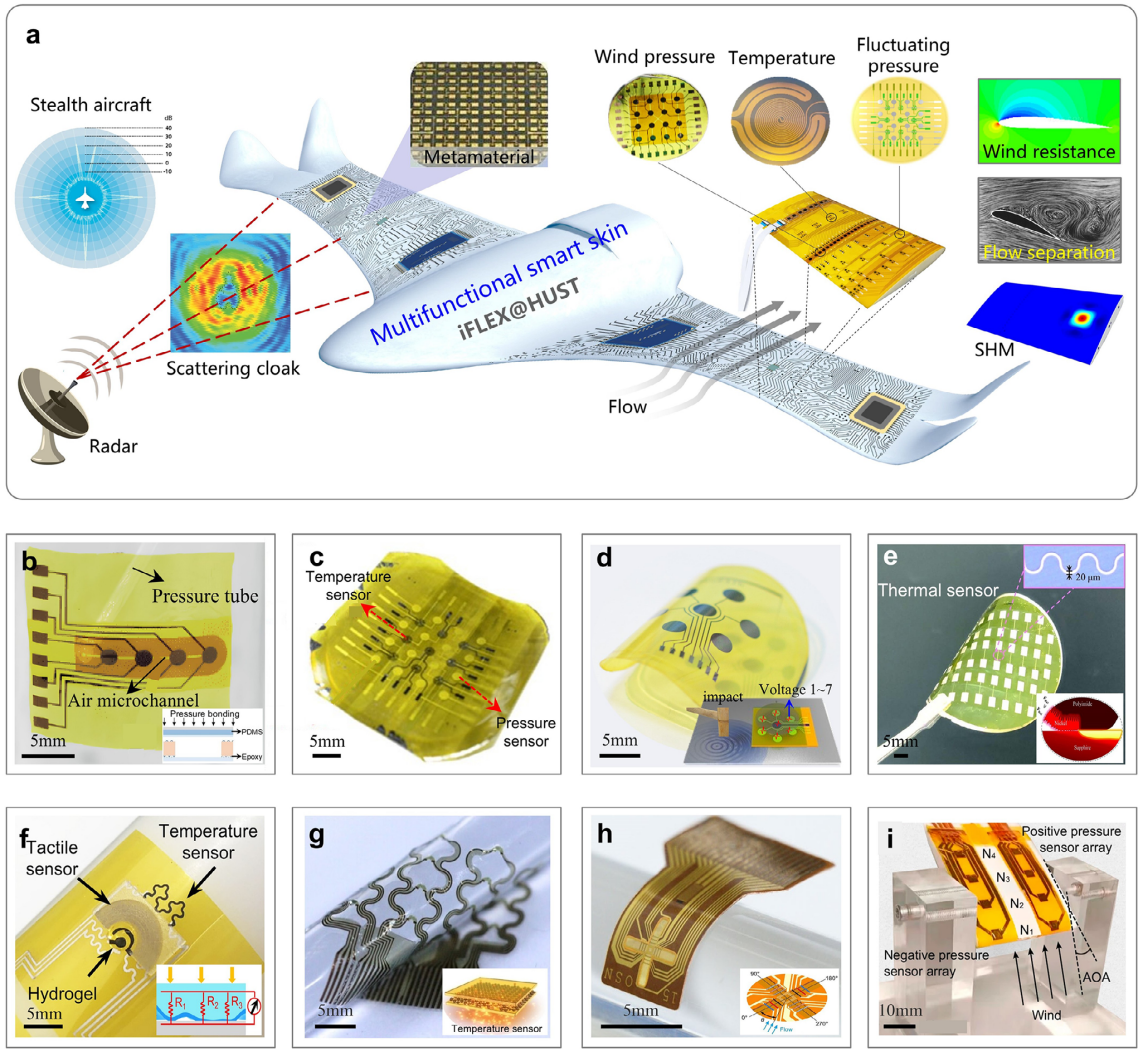
Flexible electronics for next‐generation aircraft. (a) Flexible electronics integrated as smart skins for monitoring flight‐related physical quantities. (b) A programmable flexible capacitive sensor designed for large‐area wind pressure measurement. (c) A flexible dual‐mode sensor for dynamic pressure and temperature measurement. (d) A small‐area flexible sensor array with artificial intelligence network algorithms. (e) A flexible thermal sensor with high sensitivity. (f) An AI‐driven multimodal flexible sensing system for monitoring physiological signals, tactile perception, and chemical substances. (g) A thin, stretchable array temperature sensor for high‐resolution spatial thermal imaging. (h) A single flexible heat flux sensor for evaluating multiple flight parameters. (i) A flexible smart skin for a wider range of pressure measurement. (a) Reproduced with permission [84]. Copyright 2022, Wiley. (b) Reproduced with permission [234]. Copyright 2020, Springer Nature. (c) Reproduced with permission [232]. Copyright 2020, American Chemical Society. (d) Reproduced with permission [241]. Copyright 2023, Sage. (e) Reproduced with permission [242]. Copyright 2023, Wiley. (f) Reproduced with permission [201]. Copyright 2022, AAAS. (g) Reproduced with permission [233]. Copyright 2023, American Chemical Society. (h) Reproduced under the terms of the CC‐BY 4.0 [237]. Copyright 2024, Springer Nature. (i) Reproduced under the terms of the CC‐BY‐NC 4.0 [85]. Copyright 2024, Springer Nature.

Next, Figure 8a illustrates a multifunctional smart sensing skin designed for next‐generation aircraft, which integrates aerodynamic sensing with electromagnetic cloaking capabilities [84]. This innovation represents a significant leap forward in improving both the survivability and maneuverability of future aerospace systems. Enhanced stealth capabilities are expected to become a defining feature of future aircraft. Meanwhile, unmanned aerial vehicles that can dynamically adapt their aerodynamic profiles to different flight conditions and mission requirements are emerging as another inevitable development trend [230, 231]. This multifunctional smart skin is capable of measuring various external environmental parameters such as temperature [232, 233], wind pressure [85, 234], and humidity [235, 236]. It can also monitor flight and aerodynamic states such as flow velocity, elevation angle, flutter, and stall onset [237]. Beyond surface conditions, the system can evaluate internal structural states such as stress–strain [238], damage [239], and vibration [240]. In this field, the Huang group has made a series of significant contributions, proposing a suite of innovative flexible sensors, including: (i) A programmable flexible capacitive sensor tailored for large‐area wind pressure measurement (Figure 8b) [234], (ii) A dual‐mode flexible sensor combined a PZT piezoelectric sensor with a Pt‐based temperature sensor, enabling simultaneous detection of dynamic pressure and temperature (Figure 8c) [232], (iii) A small‐area flexible piezoelectric sensor array integrated with AI‐driven network algorithms, capable of detecting impact events across areas 75 times larger than the sensor array itself (Figure 8d) [241], and (iv) A flexible thermal sensor based on a laser‐induced recrystallization process, designed specifically for measuring friction (Figure 8e) [242]. In addition, Yu et al. proposed an AI‐powered multimodal flexible sensing system capable of monitoring physiological signals, tactile feedback, and chemical substances, while achieving autonomous and intelligent decision‐making (Figure 8f) [201]. Shin et al. developed a thin, stretchable array temperature sensor capable of high‐resolution spatial thermal imaging (Figure 8g) [233]. Gong et al. proposed a single flexible heat flux sensor to evaluate multiple flight parameters, including airspeed, angle of attack, and angle of slip (Figure 8h) [237]. Wang et al. designed a flexible smart skin incorporating two ionic pressure sensors to extend the limited measurement range of conventional flexible wind pressure sensors (Figure 8i) [85].

Flexible electronics hold immense potential to revolutionize the measurement and reconstruction of curvy surfaces. However, many of their demonstrated capabilities and device prototypes remain in early‐stage development and are often limited to laboratory research or controlled demonstrations under optimal conditions. The transition toward real‐world, scalable deployment is hindered by several critical challenges: (i) Limited material selection: Intrinsically flexible and stretchable materials like polydimethylsiloxane (PDMS) and Ecoflex offer a straightforward answer for constructing flexible electronics. However, their applications have often been restricted by mechanical complexity and reliability concerns. For instance, currently available intrinsically stretchable materials generally possess a low glass transition temperature, which makes them unsuitable for high‐temperature or aerospace environments. (ii) Structural design plateau: Engineering strategies like the well‐known “island–bridge” strategy (e.g., wavy, serpentine, self‐similar, coil), enabling low‐modulus mechanics of high‐modulus materials, are widely exploited to accommodate conformal requirements. Their ultrahigh stretchability has demonstrated advances in a broad range of innovative applications. However, the sophisticated, thin, and tenuous interconnects severely dampen its usage in practical complex environments. (iii) Conformal fabrication limitations: The fabrication of flexible electronics heavily depends on 2D‐to‐3D transfer techniques, wherein devices are initially fabricated on flat substrates and then transferred onto curvy surfaces, such as transfer printings (e.g., elastomeric transfer printing, water transfer printing, and conformal additive stamp printing) and kirigami‐based and origami‐based 2D‐to‐3D designs. However, these approaches suffer from several drawbacks, such as nonuniform electrical conductivity due to stretching or compression during transfer, as well as substrate compatibility limitations, mechanical fragility, alignment issues, and fabrication complexity. As a result, the following research should be paid to: (a) Material innovation: Exploring new intrinsically stretchable materials or the functional modification of existing ones to improve the flexibility, durability, and performance of flexible electronics. (b) Flexible metamaterial electronics: Incorporating supernatural properties of metamaterials into flexible electronics for enhancing and innovating functionalities. (c) Advanced conformal fabrication: Developing fabrication strategies capable of directly patterning arbitrary electronic structures onto arbitrary curvy surfaces. Other important research avenues include wireless transmission, miniaturization, and integration.

### 3.3 Flexible Fibers

Flexible fibers are slender, elongated functional materials capable of maintaining signal transmission or sensing functionality under significant bending, twisting, and other forms of mechanical deformation. Their inherent flexibility allows them to conform seamlessly to complex surfaces, withstand external mechanical stresses, and preserve structural integrity after repeated deformation. These fibers operate by incorporating internal functional components, such as conductive nanowires or optical fiber cores, which convert geometrical deformation into measurable physical quantities (e.g., force, temperature, and strain) [248]. Based on their working mechanisms, flexible fibers can be broadly classified into two categories: flexible electronic fibers [88, 89] and flexible optical fibers [90, 91, 92]. Flexible electronic fibers are fabricated by embedding conductive materials and sensing elements (e.g., strain gauges and thermistors) either on the surface or within the fiber core [249, 250]. These fibers function by detecting changes in electrical properties, allowing them to measure mechanical deformation, pressure, and temperature. Flexible optical fibers, on the other hand, guide optical signals through flexible waveguides and convert interactions with the environment into measurable changes using optical interference techniques [90, 91]. Flexible fibers can be woven into, embedded within, or directly attached to surfaces, offering exceptional adaptability. Compared with other measurement methods, flexible fibers are particularly well‐suited for multilayer structures, internal regions, and long‐distance sensing. In addition, they provide advantages such as high breathability, lightweight construction, and enhanced comfort for wearable applications [130, 131, 132, 133, 134]. Therefore, flexible fibers are widely employed in diverse domains, including smart textiles and fabrics [243, 244, 245], wearable devices [246, 247], and soft robotics [251, 252], where mechanical adaptability and seamless integration are critical.

**FIGURE 9:**
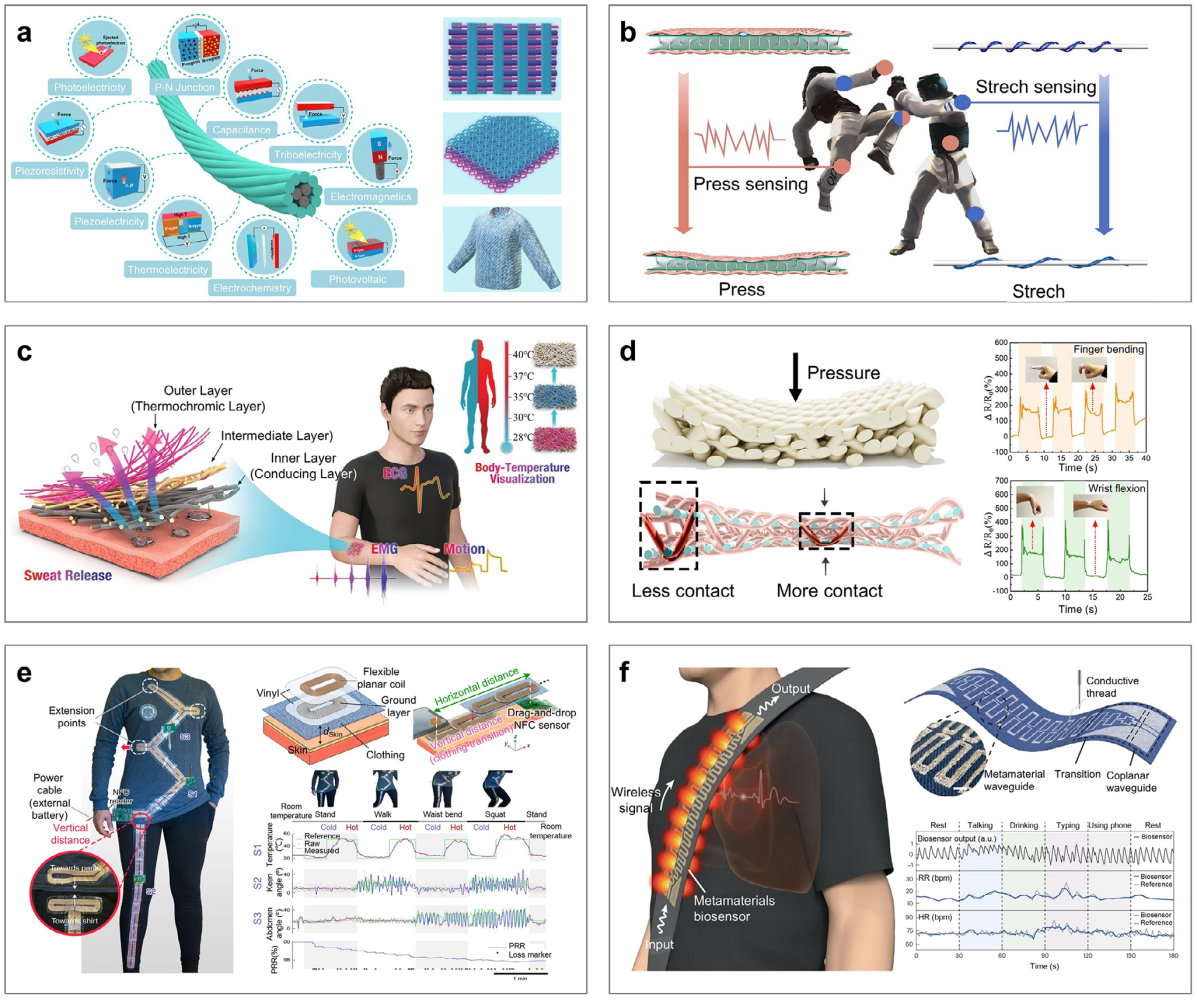
Flexible electronic fibers for physiological health monitoring. (a) Flexible functional fibers with diverse physical and chemical effects and the craft of weaving. (b) Flexible electronic textile with dual pressure and tension sensing capabilities. (c) Ultrafine fiber textile capable of real‐time monitoring of body temperature, electrocardiograms, electromyography, and skin electrical activity signals. (d) Wearable electronics based on silk textile for motion recognition and electric heating. (e) Textile‐integrated metamaterial with wireless communication. (f) A metamaterial biosensor manufactured via digital embroidery technology for capturing cardiopulmonary signals. (a) Reproduced with permission [89]. Copyright 2024, Royal Society of Chemistry. (b) Reproduced with permission [243]. Copyright 2021, Elsevier. (c) Reproduced with permission [244]. Copyright 2023, Wiley. (d) Reproduced with permission [245]. Copyright 2024, Springer Nature. (e) Reproduced with permission [246]. Copyright 2021, Springer Nature. (f) Reproduced with permission [247]. Copyright 2024, Springer Nature.

Figure 9 illustrates a range of representative applications of flexible electronic fibers, which serve as the foundational building blocks for smart textiles. Individual fibers are typically incorporated into fabrics via weaving, knitting, bonding, or similar textile manufacturing techniques. These fibers can exhibit a variety of physical and chemical functionalities, including piezoelectricity, photoelectricity, thermoelectricity, triboelectricity, electromagnetism, and electrochemistry (Figure 9a) [89]. For instance, Ma et al. developed an electronic textile with dual pressure and tension sensing capabilities, enabling the precise detection of subtle linear movements (Figure 9b) [243]. To enhance wearing comfort and mechanical robustness, Dong et al. designed ultrafine fibers with stronger adhesion, providing anti‐gravity water transport and mechanical stability under large‐scale stretching (Figure 9c) [244]. Their system further integrated a human–machine interface capable of real‐time monitoring of body temperature, electrocardiograms, electromyography, and skin electrical activity. However, the limited resilience of conductive materials relative to fabrics hindered the development of flexible fabrics. To address this problem, Chen et al. introduced a wearable electronic based on silk textile through microstructural engineering, achieving materials with high elasticity, conductivity, and durability (Figure 9d) [245]. This design supported applications such as motion recognition and electrical heating. Recently, metamaterial‐based smart textiles have attracted growing attention for their ability to manipulate electromagnetic fields in novel ways. For example, to facilitate wireless communication within near‐field networks, Hajiaghajani et al. developed a textile‐integrated metamaterial system that enabled wireless communication between multiple objects within near‐field networks, using a discrete array of magnetic induction elements (Figure 9e) [246]. Similarly, Zeng et al. proposed a metamaterial biosensor manufactured using digital embroidery technology, which can be integrated into seat belts to non‐invasively monitor cardiopulmonary signals (Figure 9f) [247].

**FIGURE 10:**
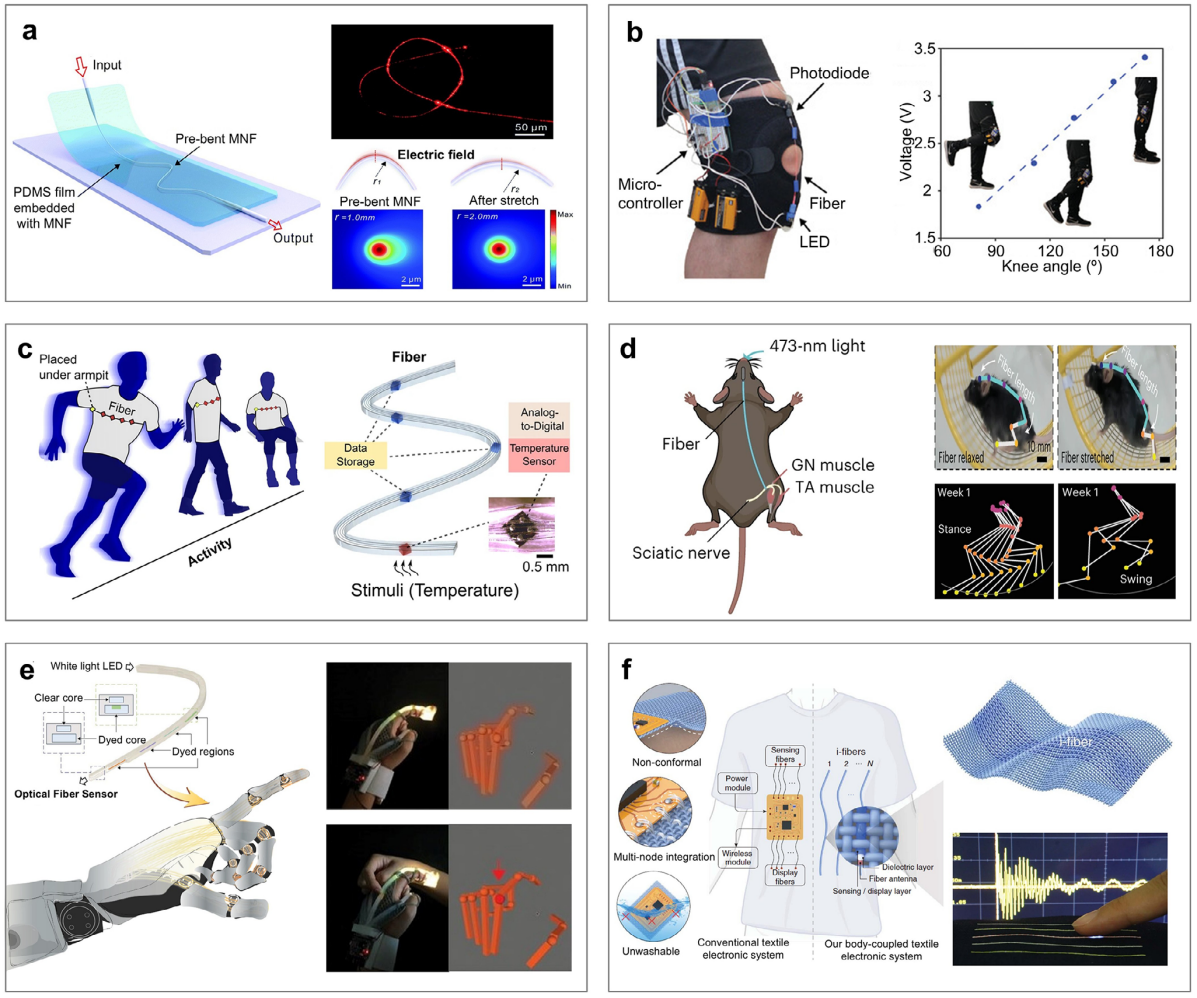
Flexible optical fibers for physiological health monitoring. (a) Optical nanofiber patch for real‐time monitoring of respiration, temperature, and motion. (b) Optical fibers sensing under large deformation. (c) Flexible optical fibers embedded with digital storage devices for long‐term physiological monitoring. (d) Invasive hydrogel fiber for light‐based nerve modulation in moving mice. (e) Flexible optic fibers for soft robotics and human–machine interfaces. (f) Microfibers integrated electronic components for wireless sensing. (a) Reproduced with permission [253]. Copyright 2020, Royal Society of Chemistry. (b) Reproduced with permission [254]. Copyright 2018, Wiley. (c) Reproduced under the terms of the CC‐BY 4.0 [255]. Copyright 2021, Springer Nature. (d) Reproduced with permission [251]. Copyright 2023, Springer Nature. (e) Optical fiber sensor: Reproduced with permission [252]. Copyright 2020, AAAS. Optical fiber in mechanical hand: Reproduced under the terms of the CC‐BY 4.0 [90]. Copyright 2024, AAAS. (f) Reproduced with permission [91]. Copyright 2024, AAAS.

Figure 10 presents a diverse range of applications of flexible optical fibers, spanning areas such as human health monitoring, biological sensing, and human–machine interfaces. Their inherent flexibility and optical sensitivity offer promising solutions for wearable and soft electronics. For instance, Pan et al. designed an optical nanofiber patch capable of conforming to the skin or other curvy surfaces, enabling real‐time monitoring of respiration, temperature, and body posture by detecting changes in the fiber's bending angle (Figure 10a) [253]. Similarly, Leber et al. fabricated soft optical fibers with tightly coupled optical and mechanical properties, allowing them to withstand extremely large strains without loss of function (Figure 10b) [254]. This advancement facilitated positioning and sensing under conditions involving significant deformation. Building on this, Loke et al. developed flexible optical fibers embedded with digital storage devices, allowing long‐term physiological monitoring and movement inference via neural network algorithms (Figure 10c) [255]. In addition to surface applications, researchers have also explored subsurface integration. Liu et al. developed an invasive hydrogel fiber capable of modulating peripheral nerves in freely moving mice using light, a major advancement for somatosensory research and optogenetics (Figure 10d) [251]. Moreover, flexible optical fibers are also being applied in soft robotics and human–machine interfaces, where movement and posture are inferred by analyzing changes in light absorption and reflection (Figure 10e) [90, 252]. However, many current smart textiles still rely on rigid components, which hinder integration, flexibility, and wearing comfort. To overcome this, Yang et al. developed integrated optical microfibers that encapsulate all required electronic components, enabling their seamless incorporation into multifunctional garments (Figure 10f) [91]. This innovation supported wireless sensing and represents a major step forward in fully integrated, flexible smart textiles.

Flexible fibers uniquely combine mechanical deformability with functional signal transmission, enabling optical and electrical sensing in a slender, highly adaptable form. As such, they represent a promising avenue for curvy surface measurement and reconstruction across applications in wearable electronics, soft robotics, and smart textiles. However, despite their significant potential, several critical challenges remain: (i) Mechanical mismatch between the functional sensing materials and the fiber substrates can lead to strain localization, signal distortion, and reduced measurement accuracy. (ii) Poor elasticity, particularly in flexible optical fibers, results in mechanical fatigue and limits performance under large‐scale or high‐frequency deformation. (iii) Trade‐off between precision and multifunctionality, while single‐mode fibers offer high accuracy but are limited to one sensing modality, multi‐mode fibers can detect multiple parameters but suffer from shorter transmission distances and lower resolution. (iv) The minimum bending radius requirement, the sharper the optical fiber bend, the more severe the light leakage and signal attenuation. To overcome these challenges, future research should focus on: (a) the development of multifunctional composite fibers with enhanced mechanical resilience, environmental stability, and signal fidelity. (b) Exploration of bio‐derived, biodegradable, or self‐healing materials to improve comfort, sustainability, and durability for long‐term applications. (c) Advanced fabrication techniques, such as nanoimprinting, surface lithography, and in‐fiber microstructure engineering, to enable scalable production of high‐performance, high‐curvature fibers. (d) Integration of photonic–electronic hybrid fiber systems to support multiplexed sensing and data transmission over large areas. (e) Robust packaging technologies that maintain performance under dynamic and repeated use conditions. Collectively, these efforts aim to realize a new generation of flexible fibers that are scalable, durable, multifunctional, and seamlessly integrable with complex, curvy, and dynamic surfaces.

## 4 Reconstruction Algorithms

Reconstruction algorithms refer to the process of establishing rational connections between discrete data points obtained from various measurement techniques to create or approximate a smooth, continuous surface that accurately reflects the geometric and physical characteristics of an object [256, 257]. The primary goal is to achieve a reconstruction that is as realistic and physically faithful as possible. Broadly, reconstruction algorithms can be categorized into two main categories: physical model‐based methods [99, 100, 101, 102, 103, 104] and data training‐driven methods [109, 110, 111, 112, 113]. The physical model‐based approach relies on the formulation of fundamental physical laws and mathematical representations of the measured data [99, 100, 101]. By incorporating governing equations (e.g., strain–strain relationships and heat flow models), along with appropriate boundary and initial conditions, a mathematical framework is constructed. Solving these equations yields the reconstructed physical fields or unknown quantities, such as displacement, temperature, or stress. In contrast, data training‐driven methods are based entirely on large datasets. These methods leverage machine learning or statistical modeling techniques to extract patterns and correlations between input measurements (discrete local data) and the output target quantities (continuous surface distribution). Through supervised or unsupervised learning, a predictive model is developed to reconstruct complex physical behaviors without explicit reliance on physical laws [109, 110, 111]. Comparatively, data training‐driven methods have become an inevitable trend in the evolution of reconstruction techniques, enabled by advances in computational power, machine learning algorithms, and the growing availability of large‐scale datasets. In contrast, physical model‐based approaches were more prevalent in earlier stages of development due to limited computational capabilities and the focus on simplified structural forms (e.g., rods, beams, and plates) [99, 100, 101, 102]. However, while data‐driven methods excel in geometric reconstruction and pattern recognition, they often fall short in capturing underlying physical laws and multi‐physical field interactions. In this regard, physical model‐based approaches remain essential for understanding fundamental mechanisms and reconstructing physical fields, especially for multi‐physical coupling fields.

**FIGURE 11:**
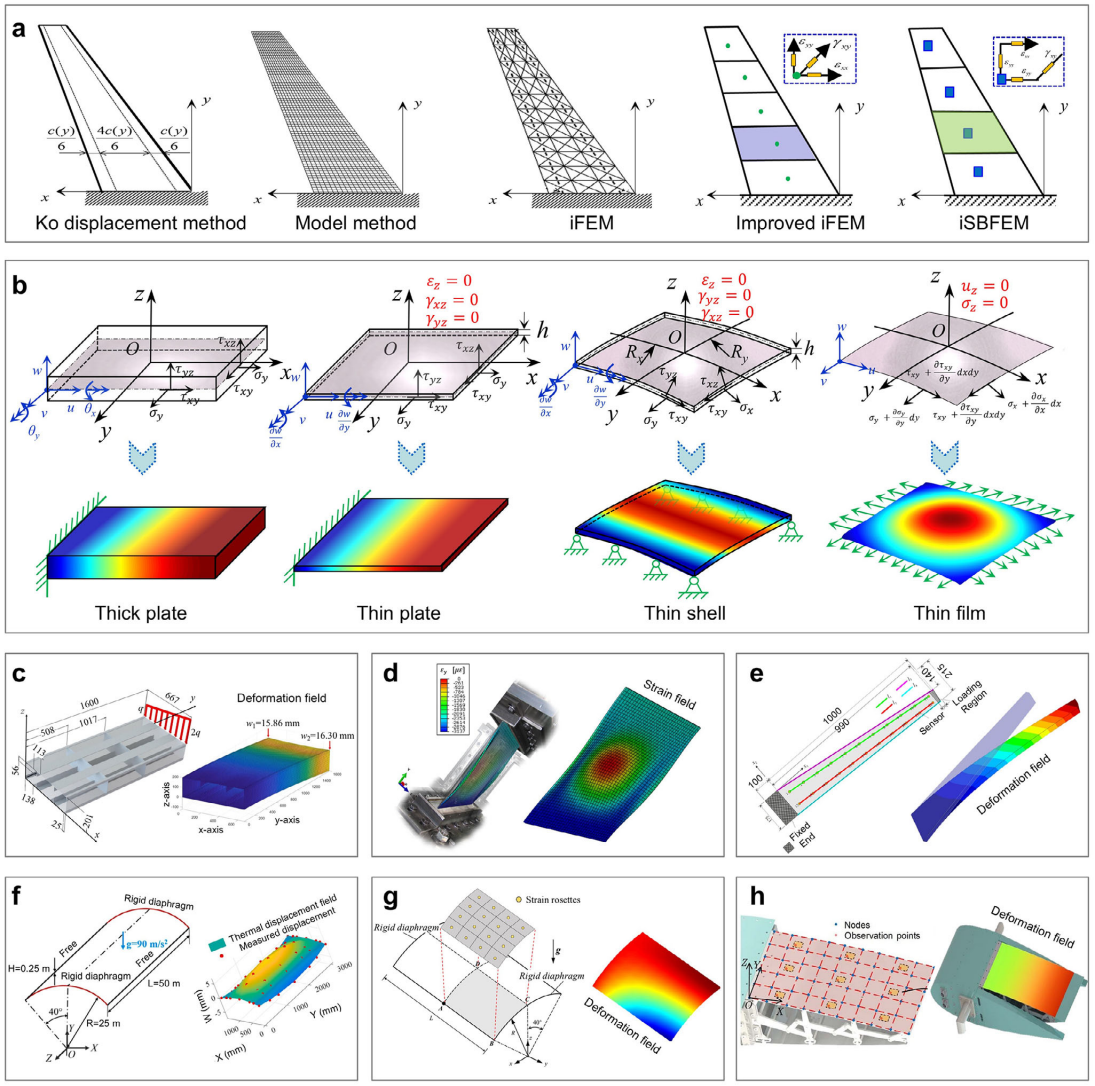
Physical model‐driven reconstruction methods. (a) Traditional physical model‐driven reconstruction methods: Ko method, modal method, iFEM, improved iFEM, and iSBFEM. (b) Deformation reconstruction of basic elements: thick plates, thin plates, thin shells, and thin films. (c) Deformation reconstruction of unswept wing box structure. (d) Global deformation perception based on smooth element analysis and iFEM. (e) Deformation sensing of sandwich materials based on refined zigzag theory and traditional iFEM. (f) Displacement reconstruction of large structures based on iFEM. (g) Combining iFEM with a tensor mixed interpolation method for deformation sensing of variable thickness structures plates. (h) A shape sensing system that combines intelligent flexible sensing films and iFEM. (a) iFEM: Reproduced with permission [101]. Copyright 2018, Elsevier. Improved iFEM: Reproduced with permission [107]. Copyright 2022, Elsevier. iSBFEM: Reproduced with permission [108]. Copyright 2024, IEEE. (b) Thick plate: Reproduced with permission [108]. Copyright 2024, IEEE. Thin plate: Reproduced with permission [99]. Copyright 2022, IEEE. Thin shell: Reproduced with permission [258]. Copyright 2024, Elsevier. (c) Reproduced with permission [259]. Copyright 2021, Elsevier. (d) Reproduced with permission [260]. Copyright 2021, Elsevier. (e) Reproduced with permission [100]. Copyright 2021, Elsevier. (f) Reproduced with permission [261]. Copyright 2024, Elsevier. (g) Reproduced with permission [262]. Copyright 2024, Elsevier. (h) Reproduced with permission [103]. Copyright 2024, IEEE.

Figure 11 presents a schematic overview of several representative physical model‐driven reconstruction methods and their typical applications, focusing primarily on the reconstruction of strain, temperature, and deformation in simple structures. As shown in Figure 11a, common physical model‐driven methods include the Ko method [94, 95, 96], modal method [97, 98], inverse finite element method (iFEM) [100, 101, 102], improved iFEM [104, 105, 106], and iSBFEM [107, 108]. The Ko method reconstructs the deflection of beam‐like structures by performing a double integration of strain, but it is strictly limited to one‐dimensional linear elastic deformations. The modal method establishes a mapping between discrete strain measurements and displacement fields based on a structure's natural vibration modes. However, this method typically requires many sensors, which can be impractical for real‐world applications. In contrast, iFEM reconstructs the deformation field by minimizing the error between measured strains and theoretically predicted strains. A key advantage of iFEM is that it does not require prior knowledge of material properties or external loading conditions [101]. Upon this, Shang et al. proposed a series of improved iFEM and iSBFEM [108], expanding the applicability to thick and thin plates, flat and curvy surfaces, and both linear and nonlinear models. As shown in Figure 11b, accurate deformation reconstruction has been achieved for a variety of structural elements, including thick plates [108], thin plates [99], thin shells [258], and thin films.

Beyond theoretical validation, physical model‐driven methods have shown effectiveness in practical structural applications. For example, Esposito et al. applied multiple reconstruction methods to an unswept wing box structure (Figure 11c) [259]. Oboe et al. introduced a smooth element analysis polynomial to fit the strain field of composite plates, facilitating global deformation perception when integrated with iFEM (Figure 11d) [260]. Subsequently, similarly, Kefal et al. incorporated refined zigzag theory into the iFEM framework, enabling accurate deformation sensing in sandwich structures (Figure 11e) [100]. To address thermal deformation reconstruction in large structures, Dong et al. proposed an iFEM based on displacement gradients, which achieved comparable reconstruction accuracy using fewer elements (Figure 11f) [261]. Extending this work, Xiao et al. integrated iFEM with a tensor mixed interpolation scheme, effectively solving shear‐locking and membrane‐locking issues in variable‐thickness plates, thereby enhancing sensing accuracy in such complex geometries (Figure 11g) [262]. Furthermore, Ji et al. demonstrated a novel approach combining intelligent flexible sensing films with iFEM, achieving displacement reconstruction using only unilateral strain measurements, significantly simplifying the sensing setup (Figure 11h) [103]. It should be noted that all the reconstruction methods discussed here are fundamentally related to the surface, even though many structures are modeled using 3D representations.

**FIGURE 12:**
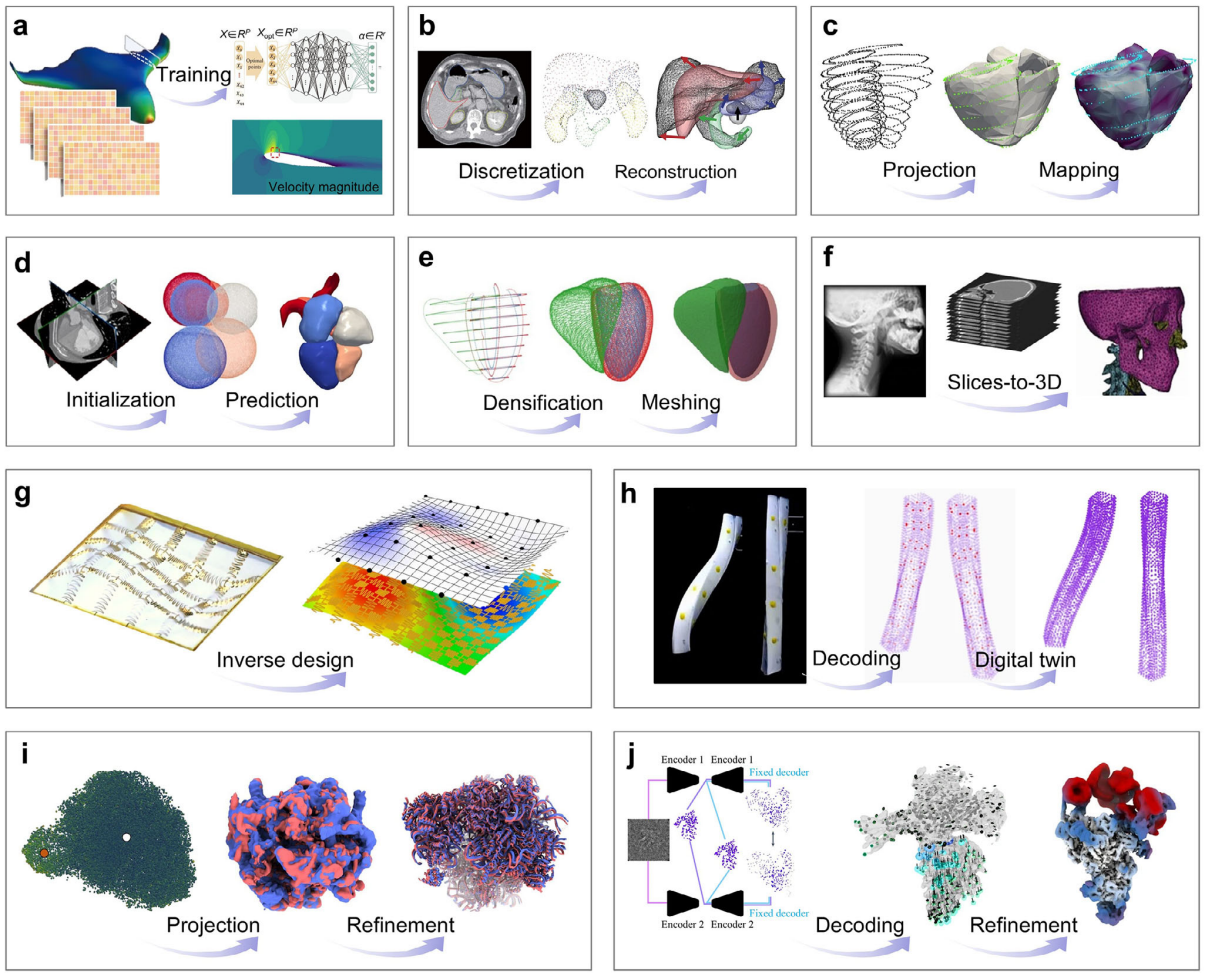
Data training‐driven reconstruction methods. (a) Real‐time reconstruction of dynamic fluid utilizing flexible intelligent skin and machine learning algorithms. (b) A region‐based depth learning algorithm for predicting pancreatic cancer displacement. (c) A hybrid graph CNN for deformation sensing. (d) CNN for 4D dynamic heart reconstruction from CT and MRI data. (e) A multi‐class point cloud completion network for heart reconstruction. (f) CNN for 3D reconstruction from X‐ray images. (g) A programmable mechanical metasurface for 3D imaging through programmable force driving. (h) A stretchable electronic skin for 3D deformation reconstruction. (i) Reconstruction of individual particle deformation. (j) Proteins deformation reconstruction. (a) Reproduced with permission [263]. Copyright 2024, Springer Nature. (b) Reproduced under the terms of the CC‐BY 4.0 [264]. Copyright 2021, Elsevier. (c) Reproduced with permission [112]. Copyright 2021, Elsevier. (d) Reproduced with permission [113]. Copyright 2021, Elsevier. (e) Reproduced under the terms of the CC‐BY 4.0 [265]. Copyright 2023, Elsevier. (f) Reproduced with permission [266]. Copyright 2022, Springer Nature. (g) Reproduced with permission [109]. Copyright 2022, Springer Nature. (h) Reproduced with permission [110]. Copyright 2023, Springer Nature. (i) Reproduced under the terms of the CC‐BY 4.0 [111]. Copyright 2023, Springer Nature. (j) Reproduced under the terms of the CC‐BY 4.0 [267]. Copyright 2024, Springer Nature.

Data‐driven reconstruction methods bypass traditional mathematical and physical modeling by directly learning nonlinear mappings between measured data and target physical quantities [109, 110, 111]. With the rapid advancement of AI, especially in fields such as computer graphics and large language models, these approaches have become increasingly prominent. This is the regime with big data, where one may not know any of the physics, and where the data training‐driven method may be most effective. By collecting, classifying, and assimilating a data deluge, extracting features, and intelligently predicting data, the data training‐driven method can obtain unknown physical relationships. However, given the breadth of this domain, and considering space limitations and the current research focus, here we highlight only representative applications of data‐driven algorithms in curvy surface reconstruction, rather than delving into algorithmic details (Figure 12). Current applied reconstruction algorithms can be broadly categorized into two types: traditional machine learning algorithms and deep learning algorithms. The former typically depend on manual feature extraction, while the latter automatically learn hierarchical features from raw input data. For example, Zhao et al. combined flexible intelligent skin with machine learning algorithms to achieve offline modal extraction and real‐time reconstruction of computational fluid dynamics (Figure 12a) [263].

In deep learning algorithms, popular reconstruction architectures include convolutional neural networks (CNNs) and recurrent neural networks (RNNs). Nakao et al. constructed a deformation library containing multiple organ shape features and proposed a region‐based deep learning model to predict pancreatic cancer displacement, enabling personalized surgical planning (Figure 12b) [264]. Similarly, Chen et al. introduced a hybrid graph CNN, which maps input images onto a template mesh and extracts spatial features to guide the deformation of the mesh, allowing for highly accurate reconstruction of anatomical structures (Figure 12c) [112]. Subsequently, Kong et al. employed CNNs to directly predict the 3D surface mesh of the heart from CT and MRI data, enabling 4D dynamic reconstruction of cardiac motion (Figure 12d) [113]. To address persistent challenges such as data sparsity, noise, and misalignment in curvy surface reconstruction, Beetz et al. proposed a multi‐class point cloud completion network with integrated data correction capabilities, demonstrating high reconstruction accuracy across thousands of clinical trials (Figure 12e) [265]. Similarly, Maken et al. utilized a series of 2D slices, such as X‐ray, to reconstruct a 3D structure using CNN (Figure 12f) [266]. Additionally, a number of statistical and optimization techniques have been employed, both individually and in combination with CNNs, to effectively achieve the transformation from 2D images to 3D reconstructions, such as statistical shape models [268], digital reconstructed radiographs [269], sequential quadratic programming [270], and principal component analysis [271].

Notably, recent advances in programmable flexible electronics and metasurfaces, when combined with AI algorithms, have opened up new frontiers in real‐time, high‐resolution curvy surface reconstruction. For example, Bai et al. introduced a programmable mechanical metasurface capable of achieving 3D imaging through programmable force driving (Figure 12g) [109]. In another example, Hu et al. proposed a stretchable electronic skin that detected spatially distributed capacitance values, enabling the mapping of complex 3D structural deformations with high fidelity (Figure 12h) [110]. Incidentally, beyond macroscopic surface applications, emerging reconstruction methods have also begun to capture microscale and nanoscale deformation phenomena. Figure 12i [111] illustrated the reconstruction of individual particle deformation, while Figure 12j [267] demonstrated the 3D conformational reconstruction of proteins, reflecting the versatility and scaling capability of modern data‐driven reconstruction techniques. Notably, most data training‐driven methods are currently limited to specific curvy surface imaging and have not yet been fully extended to physical field reconstruction. Nevertheless, their rapid evolution and recent achievements suggest that the integration of physical field reconstruction into data‐driven frameworks is becoming an inevitable trend. This trajectory is being driven by advances in computational power, deep learning algorithms, and the increasing availability of large‐scale datasets. This recognition also serves as an important inspiration and motivation for the present review. In preparing this work, we also noted related developments such as physics‐informed neural networks for solving partial differential equations [272, 273]. Such progress further supports our prediction regarding the emerging role of physics‐informed algorithms in general curvy surface reconstruction.

Reconstruction algorithms play a crucial role in achieving high‐performance reconstruction of curvy surfaces. However, (i) current reconstruction algorithms primarily emphasize geometric reconstruction or imaging focused on spatial relationships. (ii) Physical reconstruction of curvy surfaces remains limited, often addressing only simple structures and single physical quantities. (iii) Integrating physical principles into reconstruction algorithms to achieve general curvy surface reconstruction capable of simultaneously capturing geometric and physical information remains an essential yet unresolved challenge. Notably, studies of curvy surfaces have predominantly been mathematical (e.g., conformal geometry) rather than physical. (iv) Many physical mechanisms involving curvy surfaces are still not fully understood. For instance, in curvy optics or acoustics, quantitative formulas describing how curvature influences electromagnetic and acoustic wave propagation at curvy interfaces are still lacking. Similarly, quantitative analyses characterizing the conformability and stretchability of curvy surfaces in mechanics remain absent. Most existing surface solutions are restricted to simple geometries that can be represented by explicit equations, such as cylindrical, spherical, or conical surfaces. Many curvature‐related problems remain open across multiple disciplines. For instance, in curvy mechanics, there is still no unified standard for quantifying conformability and stretchability on curvy surfaces. Likewise, in curvy optics, the performance metrics and experimental standards for evaluating electromagnetic wave reflection on curvy surfaces have yet to be systematically established. In this context, (a) reconstruction algorithms are expected to evolve toward multimodal data fusion and synchronous reconstruction. For example, developing interpretable deep learning inspired by physics ensures both data accuracy and physical consistency. (b) Or improve small‐sample learning techniques to enable models to achieve effective reconstruction even with limited data. (c) Research interpretable deep learning methods or develop explanatory tools to better understand the physical significances and results of model reconstruction.

## 5 Conclusions and Outlook

Research on curvy surfaces is significant because curvature is a fundamental characteristic of the physical world, influencing phenomena across multiple disciplines, from physics and engineering to biology and materials science. Unlike flat surfaces, curvy surfaces impose geometric constraints that affect material behavior, force distribution, and functional integration, making them a rich area for investigation. Advancements in flexible electronics, soft robotics, and conformal metasurfaces rely on precise control of curvature‐related effects to ensure adaptability to dynamic surfaces while maintaining performance. In essence, research on curvature is not just about studying shapes; it is about unlocking fundamental principles that define our world and drive technological innovation. Curvy surface reconstruction serves as a key strategy for advancing this trend, aiming to recreate the “curvy world” with as much fidelity as possible. Accurate reconstruction enables researchers to model, analyze, and manipulate curvy geometries, providing deeper insights into their properties and behavior. By developing advanced computational and experimental techniques for surface reconstruction, scientists can better study curvature effects, optimize material interactions, and refine applications in areas such as biomedical imaging, structural analysis, and advanced manufacturing. Curvy surface reconstruction thus serves as a bridge between theoretical understanding and practical application, ensuring that research on curvature can be effectively translated into real‐world innovations.

**FIGURE 13:**
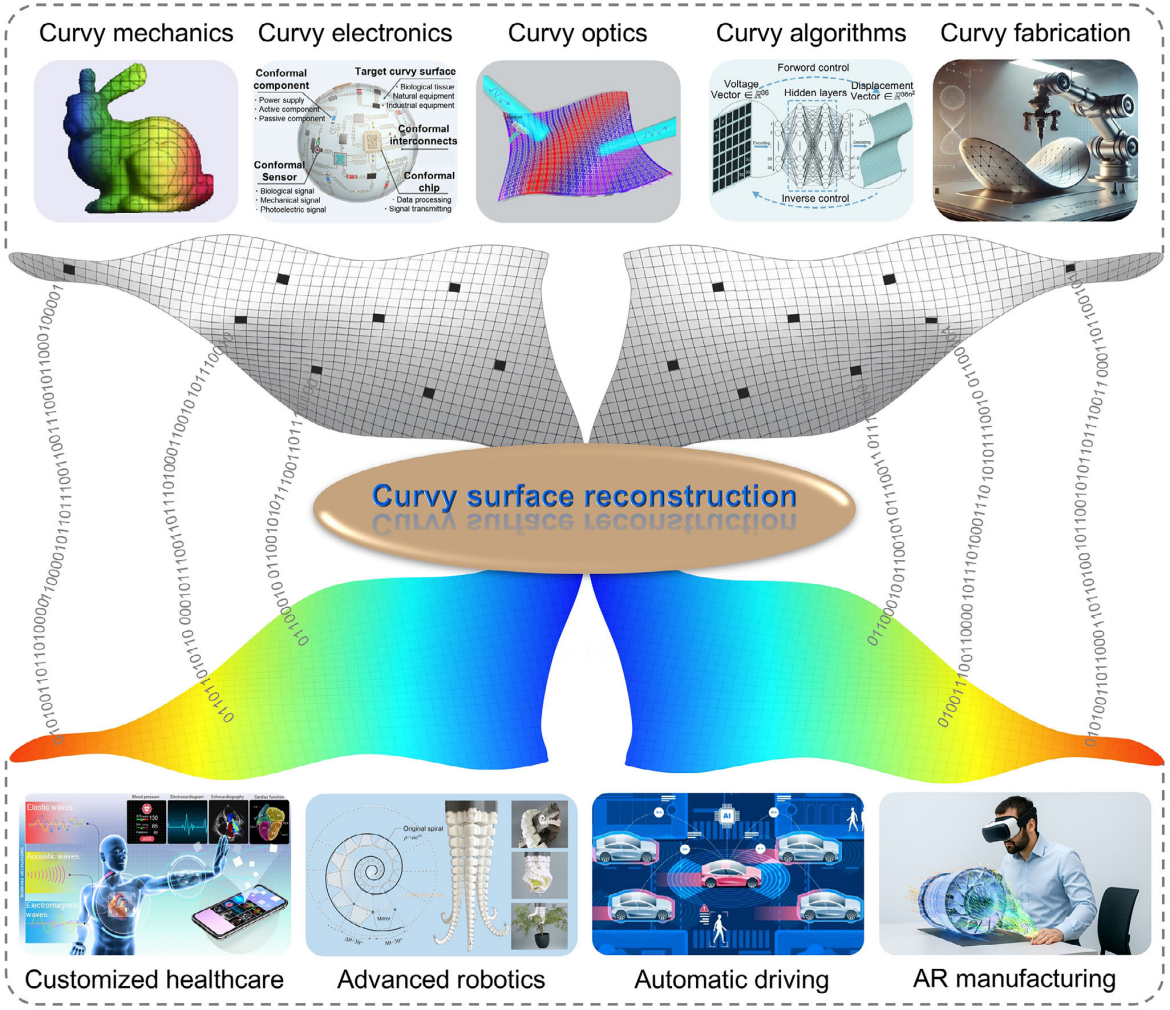
Future trends in curvy surface reconstruction. Curvy mechanics: Reproduced under the terms of the CC‐BY‐NC 4.0 [274]. Copyright 2024, Elsevier. Curvy electronics: Reproduced with permission [275]. Copyright 2025, American Chemical Society. Curvy optics: Reproduced with permission [42]. Copyright 2020, Springer Nature. Curvy algorithms: Reproduced under the terms of the CC‐BY‐NC 4.0 [276]. Copyright 2023, AAAS. Customized healthcare: Reproduced under the terms of the CC‐BY 4.0 [86]. Copyright 2023, Elsevier. Advanced robotics: Reproduced under the terms of the CC‐BY‐NC‐ND 4.0 [277]. Copyright 2025, Elsevier.

Curvy surface reconstruction is an interdisciplinary technology that draws contributions from optics, electronics, structures, sensors, algorithms, and hardware and software. Traditionally, research on curvy surface reconstruction is limited to geometric shapes, optical measurement techniques, or various reconstruction algorithms. The goal of this review is to expand the scope from special curvy imaging to general curvy reconstruction incorporating physical fields and other emerging advanced techniques, and then draw the attention of researchers from other disciplines and encourage their interests. To this end, we established a classification framework to accommodate vast variations of techniques for curvy surface reconstruction, organized and presented in a systematic manner. Representative techniques are briefly described, and illustrative figures are provided to help readers grasp their basic concepts. Selective examples of applications of curvy surface reconstruction are presented. We try to answer the following questions: What is curvy surface reconstruction? Why is curvy surface reconstruction significant? How can we get a curvy surface reconstruction? What are the future directions? This review serves as both a layman's guide to curvy surface reconstruction and a specialist's insight into the wider possibilities of curvy surface reconstruction. Then we close this discussion by identifying several important branches for future work, mainly involving more powerful measurement techniques and reconstruction algorithms, multi‐physical coupling reconstruction, and asynchronous to synchronous transition. Finally, we culminate this review in several aspects for future research (Figure 13), mainly involving conformal structures (i.e., curvy mechanics [274], curvy electronics [275]), curvy optics [42], curvy algorithms [276], curvy fabrication and applications (i.e., customized healthcare [86], advanced robotics [277], automatic driving, and AR manufacturing).

Curvy surface reconstruction entails transforming discrete, precise measurements into a continuous field, with the main challenge lying in the real‐time, in situ reconstruction of complex, flexible, and dynamic surfaces. As documented herein, curvy surface reconstruction primarily involves two steps: (i) precise measurement of discrete points on the surface and (ii) the rational connection of these points through algorithms to reconstruct a smooth, continuous surface. Thus, accurate measurement is the foundation of curvy surface reconstruction. There are many factors that characterize the technical performance of a measurement method. However, different techniques show different preferences due to the different underlying mechanisms. From an application perspective, three primary performance indices are commonly used to evaluate measurement techniques: (a) Accuracy: the maximum deviation between the measured value and the ground truth of the actual dimension of the 3D object. (b) Resolution: the smallest distinguishable portion of the object's surface that a measurement technique can resolve. (c) Speed: the acquisition rate, which is crucial for capturing moving or time‐varying objects. Beyond these primary indices, numerous other performance metrics, such as linearity, sensitivity, and reliability, can further characterize specific aspects of curvy measurement techniques. Likewise, reconstruction algorithms can be evaluated based on three primary performance indices: (a) Fidelity: the degree to which the reconstructed surface matches the actual geometry, minimizing deviation from ground truth. (b) Continuity: the ability to generate a smooth and seamless surface, avoiding artifacts, gaps, or sharp discontinuities. (c) Computational Efficiency: the balance between processing time and resource consumption, ensuring feasible reconstruction speeds without excessive computational overhead. Additional indices include robustness, scalability, and adaptability. Future measurement techniques and reconstruction algorithms should continue evolving to enhance these performance indices.

As the study of curvy surface reconstruction continues to evolve, multi‐physical reconstruction has emerged as a crucial trend. In real‐world applications, a single scenario often involves the coupling of multiple physical fields rather than just one. For instance, in robotic handshake perception, deformation, pressure, and temperature occur simultaneously. Similarly, in morphing aircraft, structural deformation, wind pressure, and temperature are concurrently present. This makes multi‐physical coupling reconstruction a rigid necessity. To address this challenge, two main strategies have been developed: (i) Multimodal sensors, where a single sensor measures multiple physical quantities, and (ii) Integration of different techniques, where each technique is dedicated to a specific physical quantity. As for multimodal sensors, although significant progress has been made, the field is still in its infancy. A major challenge is cross‐sensitivity, where the simultaneous presence of multiple inputs interferes with accurate measurements of target signals. Therefore, how to decouple sensing mechanisms plays a significant role in multimodal sensors. Future research efforts may focus on (a) Material selection, sensor design, and advanced decoupling algorithms to mitigate interference and crosstalk during signal processing, (b) Expanding beyond two physical modalities, enabling the measurement of three or more different physical quantities simultaneously [278, 279, 280], and (c) Cross‐scale conformal fabrication techniques that integrate different materials and mechanisms [281, 282]. Such as deformable sensors or functional circuits on curvy surfaces, manufacturing, and the construction of independent flexible or stretchable sensing platforms. Comparatively, integrating different techniques is more widely adopted, as it enhances adaptability to complex environments and broadens application scopes. For example, the combination of flexible electronics for contact measurement and PIV for non‐contact measurement enables the coupling of solid and fluid field measurements. Similarly, integrating flexible optical fiber with flexible electronics allows for large‐area, high‐precision, and high‐resolution deformation measurement of complex surfaces. Additionally, cross‐verification of similar functions based on different mechanisms enhances system robustness, creating synergies and reducing potential failures.

Improving asynchronous measurement to achieve synchronous measurement is a critical challenge in multi‐physical reconstruction. Synchronous measurement ensures that multiple physical quantities are captured simultaneously, maintaining temporal consistency across different data streams. This is essential for accurately reconstructing dynamic systems where physical interactions evolve in real‐time, ensuring high‐fidelity and reliable data acquisition. Conversely, asynchronous measurement, where different sensors or data sources operate with timing mismatches, can degrade measurement quality and system performance. Issues such as data misalignment, loss of temporal coherence, and phase errors introduce inaccuracies that compromise reconstruction integrity. Therefore, achieving precise temporal alignment among multiple sensing modalities is crucial for maintaining measurement reliability. However, in multi‐physical measurement and reconstruction, both multimodal sensors and integrated techniques face challenges due to differences in underlying sensing mechanisms and interference between different measurement techniques. Ensuring synchronous measurement requires a combination of strategies, including hardware precision (e.g., common clock sources, trigger mechanisms, and high‐speed data acquisition systems), software alignment (e.g., timestamp synchronization, interleaved sampling techniques, and real‐time operating systems), robust communication protocols (e.g., time‐synchronized communication standards and high‐speed data buses), calibration and compensation techniques (e.g., latency characterization, phase correction algorithms), and integrated multi‐sensor platforms (e.g., field‐programmable gate arrays, photonic chips, and photonic integrated circuits). Addressing these challenges is essential for advancing multi‐physical reconstruction and ensuring the accuracy of complex sensing systems.

Curvy fabrication is another challenging and demanding area. With rapid advances in quality and cost, we have now thoroughly entered the era of additive manufacturing dominated by 3D printing, including liquid‐based printing [283, 284], laser‐based techniques [285, 286], lithographic techniques [287, 288], as well as the more recent 4D printing trend [289, 290]. However, despite these continuous epoch‐making breakthroughs, current 3D printing methods remain largely constrained to 2D paradigms and are far from adequate to address the increasingly complex demands of curvy surfaces. At present, true curvy printing, particularly for high‐precision fabrication below 200 µm, is still rare. This significantly restricts our ability to faithfully replicate and harness the complexity of real‐world geometries. Curvy surface reconstruction is a prerequisite for curvy surface fabrication. In this process, the targeted surfaces are first modeled and then converted to STL format, followed by curvy design, curvy trajectory planning, and finally curvy fabrication. One of the major challenges impeding the advancement of curvy fabrication is the need for accurate, efficient, and robust trajectory planning on complex curvy surfaces. Existing curvy trajectory planning methods continue to suffer from limited computational efficiency, insufficient accuracy, and a lack of robustness. We are also actively committed to this research direction and have already made progress, with several of our recent works on curvy fabrication currently under submission.

Curvy surface reconstruction is expected to play a transformative role in the evolution of science and engineering beyond conventional 2D paradigms. Future research directions span multiple domains. For example, in curvy mechanics [291, 292], it enables precise mapping of stress, strain, and dynamic deformation on nonplanar structures, supporting applications such as structural health monitoring of aircraft, bridges, and biomedical implants. Curvy electronics provides the foundation for seamlessly integrating flexible sensors, circuits, and energy devices onto arbitrary geometries, advancing wearable health monitors, electronic skins, and conformal communication systems. Jian et al. proposed an automated wrap‐like transfer printing prototype for fabricating 3D curvy electronics [275], while Xue et al. introduced an ordered assembly strategy to transform 2D thin films into sophisticated 3D structures on diverse curved surfaces [293]. In curvy optics, it facilitates the design and fabrication of freeform lenses, cloaks, and metasurfaces with tailored optical responses, essential for imaging, sensing, and display technologies. Li et al. empowered reconfigurable metasurfaces to be mechanically deformable by integrating a neural network [294]. Then, Bao et al. designed integrated multifunctional metasurfaces capable of achieving asymmetric transmissions through precise amplitude–phase joint modulations [295]. In curvy algorithms [276, 296], the fusion of geometric and physical fields with deep learning and physics‐informed approaches will empower real‐time reconstruction and predictive modeling, enabling adaptive robotics, autonomous navigation, and interactive digital twins. In curvy fabrications [281, 282], reconstruction‐driven trajectory planning and multi‐material printing make high‐precision manufacturing of complex freeform devices more accessible. Looking forward, the application potential of curvy surface reconstruction is vast. For instance, in customized healthcare [86, 297], it will facilitate patient‐specific prosthetics, implants, and organ models. In soft robotics, it will allow accurate monitoring and control of deformable morphologies for intelligent locomotion and manipulation. Xie et al. implemented an electronics‐integrated soft octopus arm capable of reaching, sensing, grasping, and interacting in a large domain [298]. Then, Wang et al. presented an octopus‐inspired grasping strategy that adapts automatically to the target object's shape [277]. In autonomous driving [299, 300], it will enhance road‐surface perception and environment modeling for safer navigation. In AR‐based manufacturing [301], it will ensure precise overlay of digital designs on real‐world freeform surfaces, closing the gap between virtual models and physical production. Collectively, these opportunities position curvy surface reconstruction as a cornerstone technology in the next generation of intelligent, adaptive systems.

As an interdisciplinary field emerging from mechanics, optics, acoustics, materials science, and algorithms, curvy surface reconstruction demands both breadth and depth in research. Traditionally, research from these disciplines is more or less independently pursued with different emphases. However, recent trends call for an integrated approach that simultaneously considers measurement techniques, reconstruction algorithms, software, and hardware. This holistic strategy holds great promise for significantly enhancing the performance of curvy surface reconstruction. It is worth noting that the diversity of existing measurement techniques and reconstruction algorithms arises from the fact that no single method is universally applicable. Each technique or algorithm has its own advantages and limitations, making careful selection essential for specific applications. Readers are encouraged to weigh trade‐offs based on key performance indicators such as accuracy, resolution, speed, cost, and reliability. The field of curvy surface reconstruction is still quite young. It is our hope that our work in developing and applying curvy surface reconstruction to a variety of applications could provide some stimulation and attraction to more talented researchers from both theoretical and application backgrounds to this fascinating field of research and development.

## Conflicts of Interest

The authors declare no conflicts of interest.
